# Pancreatic Islet Protein Complexes and Their Dysregulation in Type 2 Diabetes

**DOI:** 10.3389/fgene.2017.00043

**Published:** 2017-04-20

**Authors:** Helle Krogh Pedersen, Valborg Gudmundsdottir, Søren Brunak

**Affiliations:** ^1^Department of Bio and Health Informatics, Technical University of DenmarkKgs Lyngby, Denmark; ^2^Disease Systems Biology, Faculty of Health and Medical Sciences, Novo Nordisk Foundation Center for Protein Research, University of CopenhagenCopenhagen, Denmark

**Keywords:** diabetes, data integration, protein complexes, tissue specificity, pancreatic islets, patient network biology

## Abstract

Type 2 diabetes (T2D) is a complex disease that involves multiple genes. Numerous risk loci have already been associated with T2D, although many susceptibility genes remain to be identified given heritability estimates. Systems biology approaches hold potential for discovering novel T2D genes by considering their biological context, such as tissue-specific protein interaction partners. Pancreatic islets are a key T2D tissue and many of the known genetic risk variants lead to impaired islet function, hence a better understanding of the islet-specific dysregulation in the disease-state is essential to unveil the full potential of person-specific profiles. Here we identify 3,692 overlapping pancreatic islet protein complexes (containing 10,805 genes) by integrating islet gene and protein expression data with protein interactions. We found 24 of these complexes to be significantly enriched for genes associated with diabetic phenotypes through heterogeneous evidence sources, including genetic variation, methylation, and gene expression in islets. The analysis specifically revealed ten T2D candidate genes with probable roles in islets (*ANPEP, HADH, FAM105A, PDLIM4, PDLIM5, MAP2K4, PPP2R5E, SNX13, GNAS*, and *FRS2*), of which the last six are novel in the context of T2D and the data that went into the analysis. Fifteen of the twenty-four complexes were further enriched for combined genetic associations with glycemic traits, exemplifying how perturbation of protein complexes by multiple small effects can give rise to diabetic phenotypes. The complex nature of T2D ultimately prompts an understanding of the individual patients at the network biology level. We present the foundation for such work by exposing a subset of the global interactome that is dysregulated in T2D and consequently provides a good starting point when evaluating an individual's alterations at the genome, transcriptome, or proteome level in relation to T2D in clinical settings.

## Introduction

Diabetes is a multi-tissue metabolic disease caused by defects in insulin action, insulin secretion, or both, resulting in hyperglycemia. The heritability of type 2 diabetes (T2D) has been estimated to range from 25 to 80% (Prasad and Groop, 2015). Despite that more than 120 T2D risk loci have been identified so far (Prasad and Groop, 2015) their combined effect explains only a fraction of the heritability. The unexplained heritability of complex traits is expected to mainly reside in a large number of common and rare variants across the human genome (Yang et al., 2015). Identifying the remaining variants involved in T2D through traditional single-variant association analyses will require greatly increased sample sizes compared to current studies for improving statistical power (Morris et al., 2012). Integrative systems biology approaches hold the promise to facilitate this process by considering gene products in the context of cellular networks rather than in isolation, thus improving power through the use of existing biological knowledge.

Genome-wide analyses, such as genome-wide association studies (GWAS) and studies of differential expression or methylation, often rank thousands of genes for phenotype associations. Integrating such data is a powerful way to identify genes important in the disease pathogenesis that are not identifiable in any single dataset but become evident when considering the different evidence sources collectively (Kodama et al., 2012; Pers et al., 2013). Combining such integrative evidence with protein complexes provides additional insight into the biological context and has the potential to reveal novel therapeutic targets (Lage et al., 2012).

The subset of protein complexes active in a given tissue is restricted by the tissue-specific proteome, which is important to consider because disease-associated genes have a tendency to exhibit tissue-specific gene expression in affected tissues (Lage et al., 2008). Previous studies have shown that disease-gene prioritization is improved when using tissue-specific networks compared to tissue-naive protein interaction networks (Magger et al., 2012; Ganegoda et al., 2014). Consequently, considering disease associated genes in the appropriate context is a promising avenue for making further inroads into disease understanding (Gross and Ideker, 2015). Such tissue-specific analyses are now enabled by the increasing amount of large-scale tissue and cell type specific data sets (Lonsdale et al., 2013; Kim et al., 2014; Uhlén et al., 2015), making it possible to disentangle or deconvolute tissue and cell type-specific processes.

A key diabetes tissue is the islet of Langerhans, which plays an important role in diabetes pathology. Islets are scattered around in the pancreas where they only constitute 1–2% of the total organ mass. They consist of a number of different highly specialized endocrine cell-types with the insulin-producing beta-cells and glucagon-producing alpha-cells being of the highest relevance to diabetes (Danielsson et al., 2014). Utilizing tissue-specific data, one major aim of this study was to create a pancreatic and beta-cell specific resource of protein complexes to serve as an integration scaffold in this and future studies. Previous work on tissue-specific protein interaction networks did either not include human pancreatic islets (Guan et al., 2012; Barshir et al., 2013; Basha et al., 2015) or were restricted to tissue-specific gene expression data (Bossi and Lehner, 2009; Magger et al., 2012; Greene et al., 2015). By focusing on the pancreatic islet, we supplement these resources by integrating high-confidence physical protein interaction network data with islet-specific gene expression data from both microarray and RNAseq studies, as well as protein expression from immunohistochemistry-based protein profiling.

Another major aim of the study was to identify a set of islet protein complexes that are likely dysregulated or dysfunctioning in T2D. To investigate this, we searched for complexes that were enriched for genes implicated in diabetic phenotypes through heterogeneous sources of evidence, ranging from genetic variation to methylation and gene expression in islets. The resulting complexes thus represent functional units whose perturbation can give rise to a diabetic phenotype and at the same time provide insight into the genetic heterogeneity that contributes to the pathogenesis of T2D in pancreatic islets.

## Results

### Defining a catalog of 3,692 islet protein complexes

**Figure 1:**
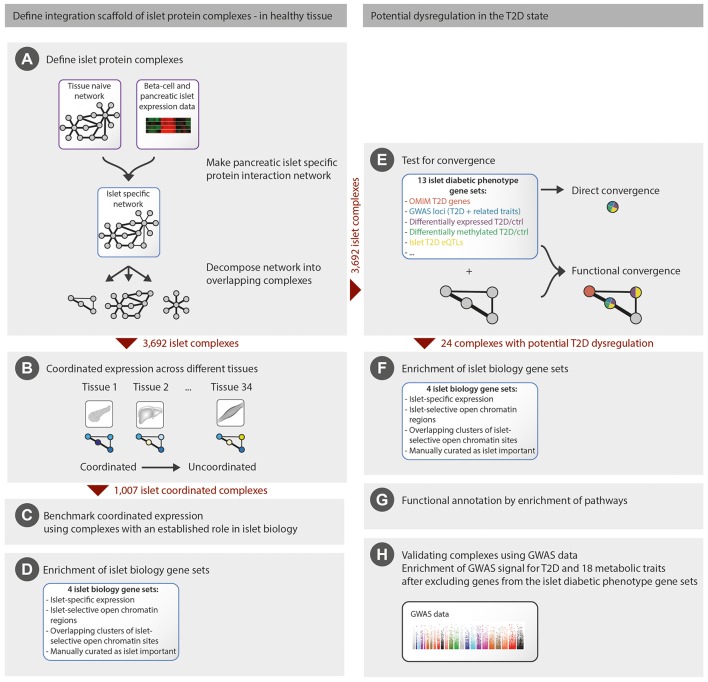
**Overview of the methodology employed**. We first generated an integration scaffold of islet protein complexes in healthy tissue **(A)** and defined a subset of complexes with coordinated expression in islets **(B)** that were further benchmarked **(C,D)**. We then identified the subset of islet complexes with potential dysregulation in the T2D state by functional convergence of 13 islet diabetic phenotype gene sets **(E)**, followed by functional annotation and validation **(F–H)**. For comparison, direct convergence of the islet diabetic phenotype gene sets was evaluated **(E)**.

We generated an islet-specific protein interaction network using gene and protein expression data combined with high-confidence protein interactions (see Section Methods and Figure 1A). This network was further decomposed into 3,692 overlapping protein complexes (10,805 genes) using the two complementary methods, ClusterOne (Nepusz et al., 2012) and spoke-hub, focusing on high internal connectivity and hub-topology, respectively (see Section Methods for details). We specifically chose network decomposition algorithms that allow for overlapping complexes as many proteins participate in multiple processes, making it difficult to decide on a single partition that closely reflects biological reality. These complexes, ranging in size from 6 to 50 proteins, captured different regions in network topology space, some being sparsely connected whereas others showed complete internal connectivity with all nodes having a physical interaction with all other nodes (Supplementary Table 1). This set of complexes represents a catalog of islet protein complexes and their constituents.

### Coordinated expression of islet protein complexes

Tissue-specific coordination of gene expression among members of a protein complex may indicate an important function of the complex in the respective tissue (Han et al., 2004; Taylor et al., 2009; Börnigen et al., 2013). To investigate the status of the islet complexes, we calculated the degree of coordinated expression of each of the 3,692 complexes across a range of 34 tissues as the normalized average Pearson correlation coefficient of interacting proteins, using data from the GTEx consortium (Ardlie et al., 2015) and the study by Nica et al. (2013) (see Section Methods for details and Figure 1B). To evaluate the importance of coordinated expression for islet relevant complexes, we defined a set of 76 benchmarking islet complexes, each constituted by 10% of genes known to be of major importance for islet function and identity (Pasquali et al., 2014; Figure 1C). These benchmarking complexes had significantly higher coordinated expression in either islets, beta-cells, or non-beta islet cells compared to the background distribution of all other complexes (MWU, *P* = 9.6 × 10^−4^, Supplementary Figure 1). These results suggest that coordinated islet gene expression of protein complex members can indicate an important role in islet biology. We therefore defined a subset of 1,007 islet-coordinated complexes where at least one of the islet tissue components (whole islets, beta, or non-beta cells) was among the three highest ranked across the 34 tissues tested (see Section Methods). Moreover, the 1,007 complexes were enriched (MWU, *P* = 2.8 × 10^−4^) for genes residing in islet regulatory regions defined as having islet-selective open chromatin in the transcription start site or gene-body (Supplementary Table 2; Figure 1D).

While these 1,007 complexes are of special interest in the context of islet function, previous work related to the cell cycle (de Lichtenberg et al., 2005) has illustrated that protein complexes can be functional even though not fully coordinated due to sophisticated, temporal regulation. We therefore included all 3,692 complexes in the further analyses on T2D dysregulation.

### Limited *Direct* overlap of islet diabetes gene sets

Having a catalog of 3,692 islet relevant protein complexes we next turned to investigate which of those were most likely to be implicated in T2D (Figure 1E). The underlying hypothesis is that complexes exhibiting pronounced convergence of genes originating from different evidence sources related to diabetes are likely to play a role in the disease.

**Figure 2:**
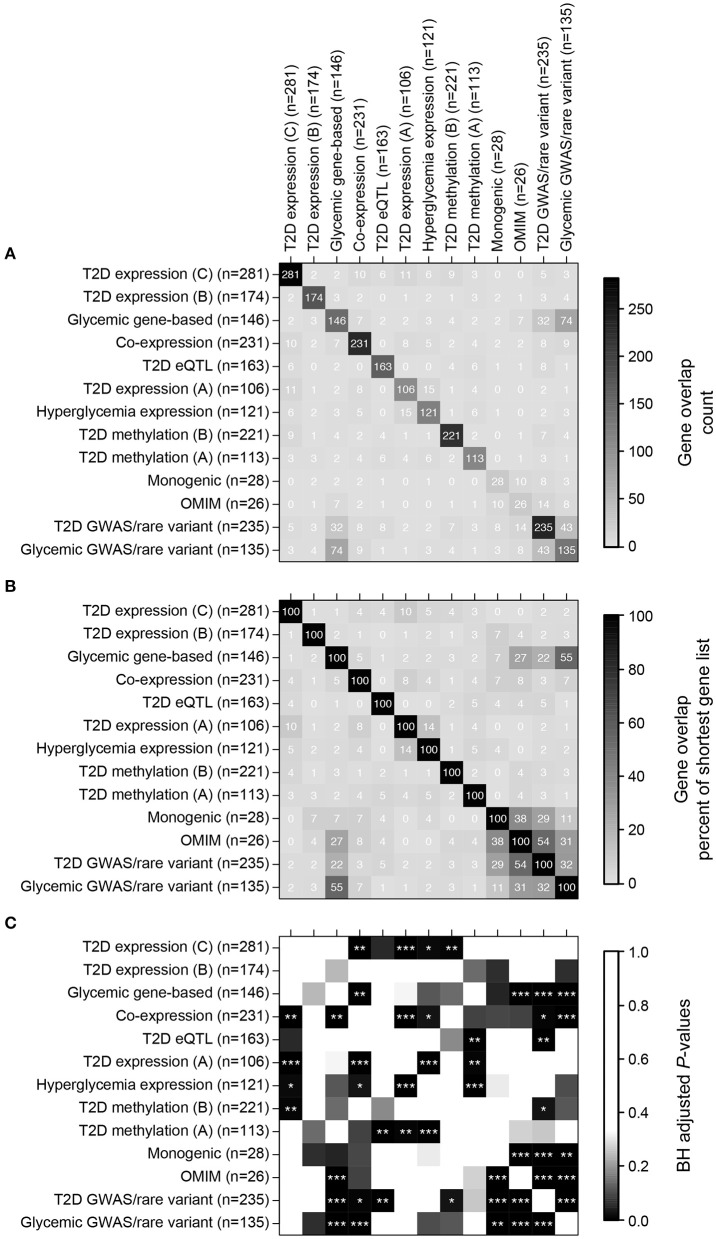
**Direct overlap of the thirteen islet diabetic phenotype gene sets. (A)** Overlap in terms of gene counts. **(B)** Overlap in terms of percent overlap relative to the size of the shortest gene sets. **(C)** BH-adjusted *P*-values for testing significance of overlap (hypergeometric test using all 22,766 genes as background), stars are as follows: ^***^P ≤ 0.001, ^**^P ≤ 0.01, ^*^P ≤ 0.05.

We thus compiled 13 sets of genes associated with T2D, monogenic forms of diabetes and related metabolic phenotypes (Table 1), hereafter termed islet diabetic phenotype gene sets. Despite all gene sets being related to diabetes, they generally showed surprisingly little direct overlap, although many pairwise overlaps were still larger than expected by chance (Figure 2). The largest overlaps, ranging from 11 to 55% relative to the size of the shortest list, were observed between gene sets based on genetic variation (Monogenic, OMIM, T2D GWAS/rare variant, Glycemic GWAS/rare variant, and Glycemic gene-based), which is to some extent expected as many genes causing monogenic forms of diabetes also harbor variants associated with T2D and glycemic traits (Bonnefond and Froguel, 2015). Twenty genes were found to be part of four or more of the 13 gene sets (Supplementary Table 3), many of which are well-known T2D susceptibility genes while others are less well-established in the context of diabetes, some of those examples are highlighted in Box 1.

**Table 1:** **Description of the thirteen islet diabetic phenotype gene sets and the four islet biology related gene sets**.

**Name**	**Description**	**References**	**# Genes (# genes in network)**
**ISLET DIABETIC PHENOTYPE GENE SETS** **GWAS LOCI AND RARE VARIANT GENES**			
T2D GWAS/rare variant	Genes in the vicinity of T2D GWAS SNPs, using a boundary of 110 kb upstream and 40 kb downstream of each gene, as well as genes harboring rare variants associated with T2D.	Morris et al., 2012; Albrechtsen et al., 2013; Flannick et al., 2014; Mahajan et al., 2014; Steinthorsdottir et al., 2014; Wessel et al., 2015	235 (162)
Glycemic GWAS/rare variant	Genes in the vicinity of GWAS SNPs (FG, BMI-adjusted FG, 2 h Glu, BMI-adjusted 2 h Glu, insulinogenic index, disposition index, proinsulin), using a boundary of 110 kb upstream and 40 kb downstream of each gene, as well as genes harboring rare variants associated with FG, proinsulin, or insulinogenic index.	Strawbridge et al., 2011; Scott et al., 2012; Huyghe et al., 2013	135 (107)
**GWAS GENES (GENE-BASED TEST)**			
Glycemic gene-based	Genes associated with FG, 2 h Glu, or proinsulin using a gene-based analysis.	Scott et al., 2012; Huyghe et al., 2013	146 (130)
**OMIM T2D GENES**			
OMIM	Genes associated with “Diabetes mellitus, noninsulin-dependent; NIDDM” in the OMIM database (accession #125853)		26 (24)
**MONOGENIC DIABETES GENES**			
Monogenic	MODY and other monogenic diabetes genes.	McCarthy, 2010; Scott et al., 2012	28 (28)
**ISLET eQTL GENES For 47 T2D SNPs (CIS AND TRANS)**			
T2D eQTL	Five cis and 176 trans eQTLs in islets, based on 47 SNPs associated with T2D.	Taneera et al., 2012	163 (129)
**GENES DIFFERENTIALLY METHYLATED IN ISLETS (T2D vs. CTRL)**			
T2D methylation (A)	Genes in differentially methylated regions that are also differentially expressed.	Dayeh et al., 2014	113 (88)
T2D methylation (B)	Genes in differentially methylated regions.	Volkmar et al., 2012	221 (169)
**GENES CO-EXPRESSED WITH 2**+ **T2D GENES**			
Co-expression	Genes that are co-expressed in islets with 2 or more of 48 T2D genes.	Taneera et al., 2012	231 (197)
**GENES DIFFERENTIALLY EXPRESSED IN ISLETS (T2D OR HYPERGLYCEMIC vs. CTRL)**			
Hyperglycemia expression	Differentially expressed genes in islets, in hyperglycemic vs. normoglycemic individuals.	Taneera et al., 2012	121 (109)
T2D expression (A)	Differentially expressed genes in islets, in T2D patients vs. controls.	Taneera et al., 2012	106 (90)
T2D expression (B)	Differentially expressed genes in islets, in T2D patients vs. controls.	Dominguez et al., 2011	174 (150)
T2D expression (C)	Differentially expressed genes in beta-cells, in T2D patients vs. controls.	Marselli et al., 2010	281 (237)
**ISLET BIOLOGY GENE SETS**			
Islet specific	Top 30 islet specific genes.	Morán et al., 2012	33 (28)
Open chromatin	Genes with islet-selective (compared to five non-islet cell lines) open chromatin in the transcription start sites or gene-body.	Gaulton et al., 2010	319 (226)
Open chromatin clusters	Genes overlapping *clusters* of islet-selective open chromatin sites.	Gaulton et al., 2010	1,512 (1,340)
Islet biology	Sixty-seven genes curated as important for islet cell identity and function, Supplementary Table 2.	Pasquali et al., 2014	67 (57)

*2 h Glu, 2 hour glucose; BMI, body mass index; FG, fasting glucose; T2D, type 2 diabetes*.

Box 1 **T2D candidate genes prioritised by direct convergence.**

The following genes were supported by four or more of the thirteen islet diabetic phenotype evidence sources, many across different levels of molecular regulation, but have not been strongly established in the context of T2D.

**The alanyl (membrane) aminopeptidase (*ANPEP*)** gene resides in a locus on chromosome 15 containing variants associated with T2D in South Asian individuals (Kooner et al., 2011) and its expression levels are furthermore associated with the T2D associated SNP rs560887 (*G6PC2* locus on chromosome 2), thus, representing a trans-eQTL (Taneera et al., 2012). In addition, the *ANPEP* gene promoter is located in a region that is hypomethylated in T2D islets (Volkmar et al., 2012), and finally the gene itself is differentially expressed in T2D beta-cells (Marselli et al., 2010). Collectively, these heterogeneous data types indicate together a plausible role of *ANPEP* in the pathogenesis of T2D in pancreatic islets. Supporting our observation, this gene has been proposed as the causal gene in this GWAS locus through a study of allelic expression profiling (Locke et al., 2015). A variant in this gene is associated with the levels of a peptide derived from the C3 complement protein that plays a role in the innate immune system (Shin et al., 2014).

**Hydroxyacyl-CoA dehydrogenase (*HADH*)** was differentially expressed in islets in three independent data sets comparing T2D patients and controls, as well as being co-expressed in islets with two or more T2D candidate genes. Mutations in *HADH* are known to cause familial hyperinsulinism (Glaser, 2013), which motivated a targeted study of common variants in the gene that however did not find any association with T2D (van Hove et al., 2006). Yet, our observations suggest that the expression of the gene is affected in pancreatic islets in T2D and that it may play a role in the disease.

**The islet expression of Family with sequence similarity 105, member A (*FAM105A*) and PDZ and LIM domain 4 (*PDLIM4*)** was associated with both T2D (Marselli et al., 2010; Taneera et al., 2012) and hyperglycemia (Taneera et al., 2012). *FAM105A* was furthermore coexpressed with the T2D genes *SLC30A8, G6PC2* and *KCNJ11* (Taneera et al., 2012) while *PDLIM4* resides in a region of the genome that was differentially methylated in islets when comparing T2D patients and controls (Dayeh et al., 2014). A variant upstream of *PDLIM4* (rs7727038) shows a nominal association (*P* = 5.2 × 10^−5^) with fasting glucose in the MAGIC consortium (Dupuis et al., 2010). Both of these genes encode for proteins with relatively unknown functions.

### Complexes showing *Functional* overlap of islet diabetes gene sets

We next investigated if the 13 islet diabetic phenotype gene sets functionally converged on any of the 3,692 islet protein complexes, by calculating the combined enrichment for the 13 gene sets for each complex (Figure 1E). We found that the 1,007 complexes with coordinated expression in islets were enriched for small *P*-values (MWU, *P* = 1.66 × 10^−5^) and we furthermore observed significant convergence of the islet diabetic phenotype gene sets in 24 complexes (9 coordinated, 15 un-coordinated) after adjusting for multiple hypothesis testing (BH adjusted *P* < 0.05; Supplementary Table 4, Data Sheet 1). All of these 24 complexes contained one or more gene supported by genetic evidence (GWAS, rare variants or monogenic forms of diabetes), suggesting that the majority are likely to play a causal role in the development of T2D (Supplementary Table 4).

The 24 complexes were additionally enriched for genes in all four islet biology gene sets (Supplementary Table 2; Figure 1F), suggesting an important role in pancreatic islets. The complexes largely showed limited gene-overlap (Supplementary Figure 2), which indicates that they span different parts of the islet interactome.

**Figure 3:**
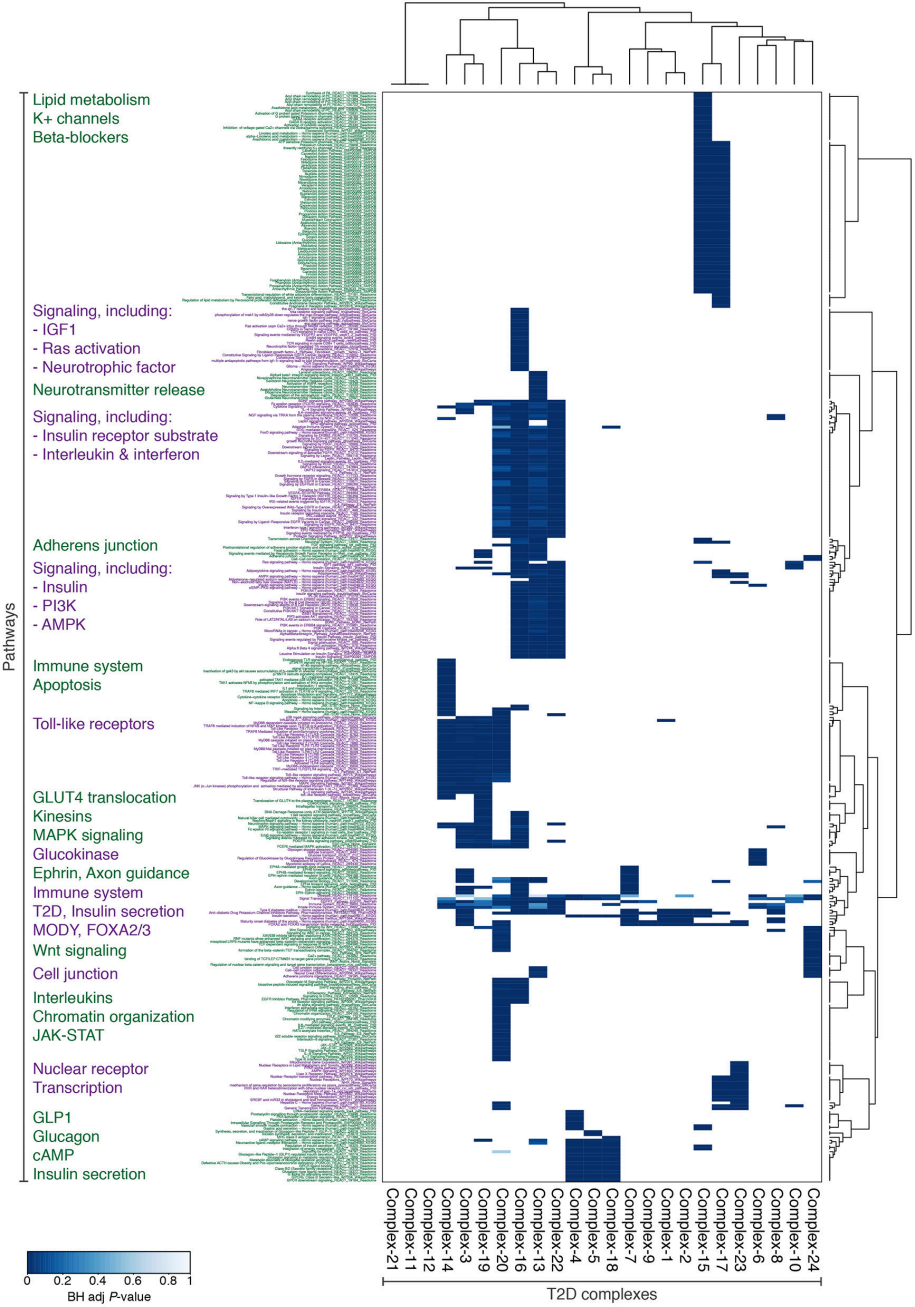
**The 24 complexes with potential T2D dysregulation are enriched for diverse and relevant functions**. Subset of Consensus PathDB-pathways, for which at least one protein complex is enriched with BH-adjusted *P* < 0.001. The pathways and complexes are clustered with Ward's hierarchical clustering using an asymmetric binary similarity measure.

We next investigated the biological functions of the 24 diabetic phenotype associated complexes (Figure 1G), and found that the complexes segregate into functional distinctive groups based on their pathway enrichment patterns (Figure 3). A number of these groups were characterized by molecular processes well-known to be dysregulated in diabetic islets—such as potassium channels, glucokinase, incretin signaling, and Wnt signaling—while others were enriched for processes less established in the islet pathogenesis of T2D, such as insulin-, interleukin-, and ephrin-signaling, cell and adherens junctions and neurotransmitter release. Interestingly, seven of the 24 complexes contained one or more target of FDA-approved drugs, many of which are not anti-diabetic agents (Data Sheet 1).

### Leveraging the complexes to propose novel T2D genes

The 294 genes constituting the 24 complexes are all interesting in the context of diabetes (Supplementary Table 5). Obviously, many of them already have an established role in T2D. By contrast, the subset of 217 genes that were not part of any of the 13 islet diabetic phenotype gene sets comprise an interesting set for further prioritization. In particular, we identified six genes (*MAP2K4, PDLIM5, PPP2R5E, SNX13, GNAS*, and *FRS2*) of high interest as novel T2D associated genes, as they all have additional support for being of relevance for islet biology or function from the islet biology gene sets and furthermore SNPs in the vicinity of these genes are associated with T2D or glycemic traits with *P* < 1 × 10^−4^ (Table 2).

**Table 2:** **Plausible novel T2D genes prioritized from the complexes with potential T2D dysregulation**.

**Gene symbol**	**Gene name**	**# Islet diabetic phenotype gene sets**	**# Islet biology gene sets**	**Minimum *P*-value for associated SNPs**	**Corresponding GWAS trait**
*MAP2K4*	Mitogen-activated protein kinase kinase 4	0	1	7.83 × 10^−6^ (rs929441)	AUCIns/AUCGluc
*PDLIM5*	PDZ and LIM domain 5	0	1	9.87 × 10^−5^ (rs17021900)	Fasting glucose
*PPP2R5E*	Protein phosphatase 2, regulatory subunit B, epsilon isoform	0	1	7.05 × 10^−5^ (rs10151995)	Fasting glucose
*SNX13*	Sorting nexin 13	0	1	4.02 × 10^−6^ (rs2723517)	HbA1c
*GNAS*	GNAS complex locus	0	1	4.73 × 10^−5^ (rs6026565)	Fasting glucose, Manning
*FRS2*	Fibroblast growth factor receptor substrate 2	0	1	9.76 × 10^−6^ (rs12425398)	Fasting glucose, Manning

*Genes are prioritized if they, besides being part of a protein complex showing potential T2D dysregulation, are part of at least one of the four islet biology gene sets and harbor at least one SNP with P < 1 × 10^−4^ in one or more of the 19 GWAS described in Supplementary Table 6. Only the best SNP P-value and corresponding GWAS trait are shown. To focus on novel T2D genes, genes in any of the 13 islet diabetic phenotype gene sets are excluded*.

Interestingly, after our analysis was completed, a targeted study of variants in the *PDLIM5* gene reported an association with T2D (rs11097432, *P* = 1.07 × 10^−3^; Owusu et al., 2017). Additional support for the prioritized genes emerges from the recent wave of single-cell transcriptomics studies of human islets that were published after our analysis was finished (Segerstolpe et al., 2016; Wang et al., 2016; Xin et al., 2016; Lawlor et al., 2017). Remarkably, *GNAS* is among the 11 genes showing consistent differentially expression in diabetic cell types (compared to non-diabetic) with same direction of effect in beta-cells (higher in T2D) in the first three studies and, furthermore, one (of 41 genes) found by both Lawlor et al. and Segerstolpe et al. with same direction of effect in alpha-cells (lower in T2D; Lawlor et al., 2017). In addition, Xin et al. (2016), reports *GNAS* to be abundant in all four major islet endocrine cell types (alpha, beta, delta, PP) in both non-diabetic and T2D donors (but not significantly differentially expressed). *SNX13* also exhibits cell type specific differential expression in T2D, being lower in delta cells of diabetic donors (fold change = −13.02, FDR = 4.93 × 10^−2^; Xin et al., 2016). Whole islet gene expression (profiled with microarrays and RNA-seq) is further nominally associated with lower HbA1c levels for both *GNAS* (*P* = 2.14 × 10^−3^, FDR = 4.30 × 10^−2^) and *SNX13* (*P* = 1.61 × 10^−2^, FDR = 1.02 × 10^−1^; Fadista et al., 2014). In mice, disruption of the G protein α-subunit (one of the *GNAS* gene products) maternal (but not paternal) allele leads to severe obesity, hypertriglyceridemia, impaired glucose tolerance and insulin resistance (Xie et al., 2008). Together, these observations add support for the genes being important for shaping the diabetic phenotype in one or more islet cell types.

Both *MAP2K4* and *GNAS* are known to be involved in pancreatic cancer [Cancer Gene Census (Forbes et al., 2017) and Intogen (Gonzalez-Perez et al., 2013) databases]. The *MAP2K4* gene encodes the mitogen-activated kinase kinase (MKK)4, which constitutes a part of the apoptotic-effect mediating MEKK1-MKK4-JNK pathway (Xia et al., 1995) and is inhibited in pancreatic beta-cells by the glucagon-like peptide-1 analog exending-4, resulting in protection from palmitate-induced apoptosis (Natalicchio et al., 2013). *MAP2K4* is furthermore a proposed tumor suppressor gene and is significantly under-expressed in metastatic compared to benign pancreatic endocrine tumors (or islet cell tumors; Couvelard et al., 2006). These results point to an important role of *MAP2K4* in the survival of pancreatic islet cells, which is a process central to the etiology of both diabetes and pancreatic carcinomas. Further studies of the potential dual role of *MAP2K4* and *GNAS* might help elucidating the molecular basis for the complex bidirectional relationship observed between diabetes and pancreatic cancer (Li, 2012).

### Verification of potential T2D dysregulation of complexes using GWAS data

As the 24 complexes were enriched for genes associated with diabetes and glycemic traits (input genes), it is likely that their disruption gives rise to these phenotypes. Thus, the remaining (non-input) genes in the complexes have a high likelihood of also contributing to the same traits. We tested this hypothesis by investigating the enrichment of GWAS signals for T2D and glycemic traits from the DIAGRAM and MAGIC consortiums in each of the 24 complexes (Figure 1H). Using the Meta-Analysis Gene-set Enrichment of variaNT Associations (MAGENTA) tool to test the enrichment, we identified 30 significant (*P* < 0.05) complex-trait combinations, spanning 15 complexes and 13 traits, of which 25 remained significant after excluding any genes that were used as input in the corresponding gene sets used for discovery of the complexes (Supplementary Figure 3). The last definition was applied to avoid any circularity, as the different GWAS might be the source of the association leading to the gene being in the islet diabetic phenotype gene sets that were used to define the 24 complexes with potential T2D dysregulation. These results indicate that the non-input genes in the complexes indeed harbor variants that are associated with the same phenotypes, although not so strongly that they could be discovered by the GWAS analysis alone.

**Figure 4:**
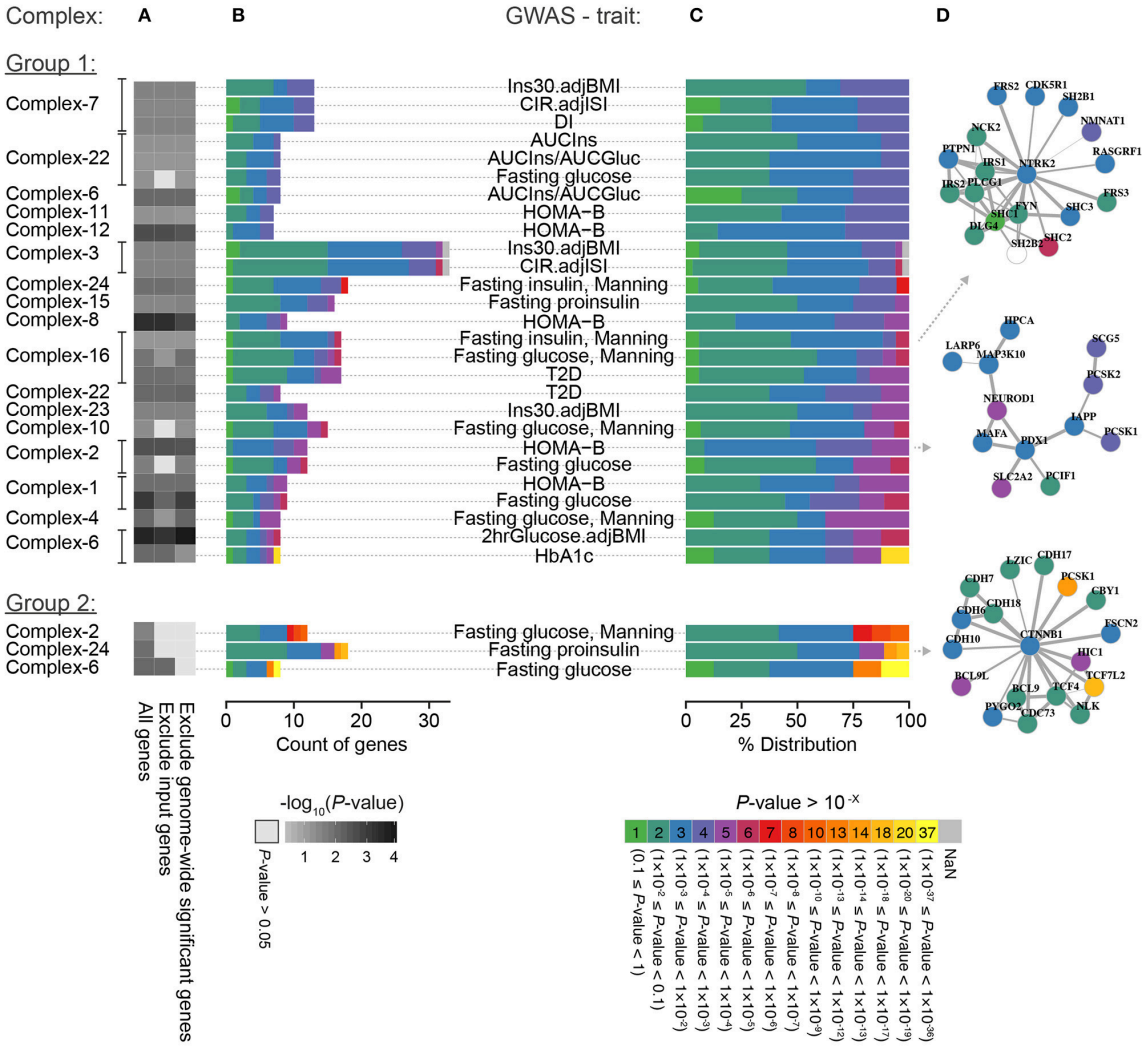
**Breakdown of significantly enriched complex-trait combinations. (A)** The enrichment of GWAS signals for each of the 30 significant complex-trait combinations when including all genes, excluding input genes, and excluding genes with genome-wide significant association in the given GWAS (see Section Methods for details). The genes in each complex-trait combination are colored based on *P*-value (i.e., minimum *P*-value for the SNPs mapping to the respective gene) partitioned into factor-10 groups; **(B)** actual count and **(C)** percentage distribution of gene *P*-values within a complex in the GWAS for the given glycemic trait. **(D)** Example of complexes.

We further investigated if the GWAS enrichment within the complexes was driven by many genes in loci with modest associations converging in the same functional context or mainly by one or a few genes with low *P*-values (minimum *P*-value for the SNPs mapping to the respective genes). We therefore repeated the analysis after excluding all genes with genome-wide significant *P*-values (*P* < 5 × 10^−8^) in the respective GWAS and found that the enrichment for 27 out of 30 complex-trait combinations remained significant (Figure 4, Group 1). This suggests that the majority of the complexes represent examples where many small effects collectively perturb their function, leading to a molecular phenotype that gives rise to disturbed glucose homeostasis. All of the three complex-trait combinations that became non-significant (Figure 4, Group 2) contained one or more gene with a genome-wide significant signal (*P* < 5 × 10^−8^), indicating that these genes were the main driver of the enrichment.

### The nature of the evidence sources behind the enrichment

**Figure 5:**
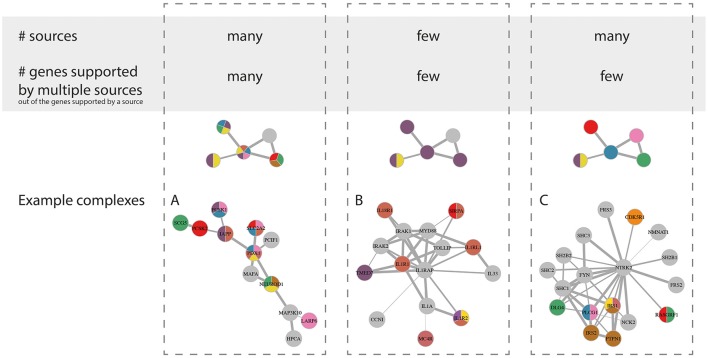
**Highlevel grouping of complexes by nature of evidence driving their enrichment**. Schematic visualization (top) and representative examples (bottom) for the three overall groups. The fourth theoretical category with few sources but a high percentage of genes supported by multiple sources is excluded here, as we did not observe any good examples. Group A, Complex-2; group B, Complex-14; and group C, Complex-16.

The 24 diabetic phenotype associated complexes could further be characterized by the diversity of supporting data driving their enrichment, such as the proportion of genes in the complex supported by multiple gene sets and the total number of gene sets supporting each complex. More specifically, we observed three notable trends (Figure 5) where the enrichment of a complex was mainly driven by (a) genes supported by multiple sources each, (b) genes supported by one or few sources each and few in total, and (c) genes supported by one or few sources each but many in total. A representative example from each of these three groups of complexes is shown in Figure 5. In group (A), the complex Complex-2 consisted of many genes that are associated with multiple diabetic phenotypes each and are well-established in the context of diabetes, including the transcription factor *NEUROD1*, which is required for normal beta-cell development, and *SLC2A2*, which encodes GLUT2—the main glucose sensor in rodent beta-cells (but not human; McCulloch et al., 2011). Furthermore, the complex contained a number of genes directly involved in insulin transcription and secretion, such as the insulin regulating transcription factors *PDX1* and *MAFA, PCSK1* and *PCSK2*, which are known to localize with insulin in islets, *IAPP*, which is co-secreted with insulin and *SCG5*, which is a marker of insulin secreting tumors. Interestingly, the *LARP6* gene in the complex was included in the islet diabetic phenotype gene sets because of its proximity to the fasting proinsulin associated SNP rs1549318 (Strawbridge et al., 2011). Its presence in the complex suggests that *LARP6* may play an important role in beta-cell function and insulin secretion. In line with the function of the genes in the complex, the overall complex was enriched for genetic associations with HOMA-B based on MAGIC data.

Complex-14 is an example from group (B), where the enrichment was driven by genes mainly supported by the same gene set (5/7 genes), namely the “Hyperglycemia expression” data. The additional supporting gene sets were mainly from gene expression or methylation sources, while it only contained one gene (*MC4R*) supported by genetic evidence that was only weakly connected to the remainder of the complex. Furthermore, no enrichment was found for low SNP *P*-values in MAGIC and DIAGRAM data. This might as such be an example of a complex that is rather involved in a response to the diabetic state in the islets than playing a causal role. This is fitting with it being mainly composed of interleukins and toll-like receptors and enriched for inflammatory response and apoptosis pathways that have a clear relevance to the beta-cell mass deterioration in T2D pathogenesis.

Finally, Complex-16 is an example of a complex where the enrichment was supported by multiple sources, but few consensus support genes. Such complexes are interesting because they could not have been revealed using any data type alone, but constitute a functionally related group of genes that are identified by multiple types of diabetes-associated evidence. Complex-16 was strongly enriched for brain-derived neurotrophin (BDFN) signaling. BDFN has indeed been shown to affect the histological organization of beta and non-beta cells in the pancreatic islets (Yamanaka et al., 2006). This complex was furthermore enriched for GWAS signals for fasting glucose levels, fasting insulin levels and T2D.

## Discussion

To harvest the power of data integration, we have brought together results from genetic studies of islet-relevant phenotypes and human islet studies spanning different levels of molecular regulation. We identify 24 protein complexes with strong supporting evidence for being implicated in diabetes pathogenesis in pancreatic islets and show how they are enriched for multiple modest effects of genetic variants associated with glycemic traits. Furthermore, we specifically prioritize ten candidate genes for T2D, of which six are novel, based on the investigation of either direct or functional convergence of the evidence sources. Additionally, we compose a set of 3,692 islet protein complexes that can serve as an integration scaffold for future studies.

By comparing the direct overlap between the heterogeneous islet diabetes-related gene sets we identified genes such as *ANPEP* and *HADH* that are currently not well-established as diabetes susceptibility genes but had consensus support across evidence sources. These observations highlight that such a straightforward data integration approach is able to pinpoint potentially new disease genes. Apart from these few, but interesting, examples of genes that were part of multiple gene sets, the generally limited direct overlap between the gene sets emphasizes the necessity of integrative systems biology approaches focusing on functional entities rather than single genes for further understanding of the dysregulation and dysfunctioning occurring in diabetic islets.

Previous work on congenital heart disease (Lage et al., 2012) has shown similar results, where a limited overlap was observed between genes identified in different types of genetic studies whereas they converged significantly in protein networks related to heart development. Here we extended this approach to T2D, where we found the prioritized complexes to mainly be involved in signaling cascades, immune functions, apoptosis and cell-cell communication in addition to the expected insulin secretion pathway. We thus show that these particular molecular mechanisms are consistently supported by complementary types of molecular data from human islets to form a major component of the T2D etiology. These results reduce the many previously observed pathways related to T2D pathogenesis in human and animal islets from single omics studies to a set of highly credible pathways.

A previous systems genetics study of the T2D state in human islets (Taneera et al., 2012) identified a set of 20 genes that collectively explained a significant portion of HbA1c variation. Here we add to those results by combining multiple independent data sets to identify nine additional T2D candidate genes that likely play a role in pancreatic islets. Furthermore, we prioritized specific protein complexes and their associated pathways that provide biological insight into T2D pathogenesis.

The majority of the 24 protein complexes found in this study were enriched for modest GWAS signals, suggesting that multiple small effects collectively perturb the complexes and give rise to variation in glycemic phenotypes. We thus provide insight into the mechanisms by which common genetic variation translates into a disease phenotype, which supports that the multifactorial genetic architecture of complex traits is constituted by a large number of variants disrupting cellular networks (Schadt, 2009).

An advantage to investigating functional convergence on protein complexes is that not all genes in the complex need to have prior diabetes-related evidence for the complex to be significant. Consequently, this approach concurrently prioritizes genes without prior diabetes-related evidence, but whose products interact with other diabetes relevant proteins in the islet, such as the six T2D candidate genes highlighted in Table 2. Furthermore, complexes containing both genes from GWAS loci and genes supported by other evidence sources, provide support for the GWAS gene mediating the signal in that locus, such as *LARP6* in the complex Complex-2 that resides in a proinsulin associated GWAS locus. Lastly, the complexes provide a functional context for the disease genes. Many genes naturally participate in several functions, reflected by the overlap of many of the complexes. For such multifunctional genes, the approach outlined here prioritizes the subset of disease relevant complexes and thus the disease relevant functions.

A major goal for T2D and other common diseases is to identify causal pathways and network modules underlying disease pathogenesis to enable precise risk prediction and development of new therapeutic strategies (McCarthy, 2015). Furthermore, such pathways and network modules need to be identified in a tissue-specific context (Gross and Ideker, 2015). Here we provide causal network modules for T2D in the form of tissue-specific protein complexes that provide more biological insight into the disease pathogenesis than disease genes in isolation and furthermore form a basis for integrating person-specific genetic, transcriptomic, or proteomic profiles in a clinical setting. Dissecting these complexes can moreover reveal new drug-targets, such as genes interacting with targets of currently used anti-diabetic medications, genes supported by multiple evidence sources or their more druggable interaction partners. Furthermore, complexes that contain targets of FDA-approved drugs may highlight opportunities for drug repurposing in the search for new diabetes treatments.

## Methods

### Construction of a pancreatic islet-specific protein interaction network

Previous tissue-specific protein interaction networks mainly fall into three categories: node-removal, where interactions between proteins absent in the given tissue are excluded (Bossi and Lehner, 2009; Barshir et al., 2013; Basha et al., 2015), edge-reweight, where interactions between absent proteins are down-weighted (Magger et al., 2012), and data-driven Bayesian methodologies (Guan et al., 2012; Basha et al., 2015; Greene et al., 2015). Here we created both an edge-reweighted as well as a node-removal islet-specific protein interaction network, to accommodate downstream network analysis approaches that did or did not consider edge-weights, respectively.

The islet-specific protein interaction networks were constructed by pruning high confidence protein interaction from an updated version (2014) of the InWeb database (Lage et al., 2007; 14,536 proteins with 337,951 high-confidence interactions) using the data sets described in Supplementary Table 7. More specifically, for the node-removal protein interaction network, genes not passing the specified cutoffs in all of the data sets were considered less likely to be expressed in pancreatic islets and thus removed from the pruned islet network. For the edge-reweighted protein interaction network, lowly expressed genes were not removed but instead the confidence score of their interactions were down-weighted using the approach proposed by Magger et al. (2012):

$$\begin{array}{l} w_{ij}^{\prime }=w_{ij}{\;}^{*}\;rw^{n} \end{array}$$

where *w*_*ij*_ is the original edge weight between protein *i* and *j, n* is the number of lowly expressed genes in the tissue constituting the interaction (i.e., {0,1,2}), and *rw* is the probability that a gene is expressed in the tissue even though it does not pass the cut-offs listed below, which was chosen to be 0.1.

If genes were not covered by any of the data sets—or in the case of the Human Protein Atlas (HPA) data, annotated with uncertainty—a benefit-of-the-doubt approach was applied where such genes were considered present.

### Included data-sources

Tissue-specific protein expression profiles based on immunohistochemistry using tissue microarrays were obtained from the HPA version 13, 11/6-2014, downloaded on 10/3-2015 from www.proteinatlas.org, with Ensembl version 75.37 (Uhlén et al., 2015). Proteins were categorized as present, absent, or uncertain based on the reliability and level of their expression value. Specifically, proteins with supportive expression values were categorized as absent if they were not detected and otherwise as present if they had low, medium or high expression values whereas proteins with uncertain expression values were categorized as uncertain.

Microarray gene expression data from the GNF Tissue Atlas (GNF) (GEO: GSE1133) was downloaded from BioGPS (http://biogps.org/downloads/; Su et al., 2004).

### Defining topology-based complexes within the network

Many different methods with different objective functions have been proposed for defining clusters of genes in protein interaction networks. Here we applied two complementary approaches; one aiming at identifying tightly connected genes, and one centered on spoke-hub complexes as often applied in previous work (Lage et al., 2007; Börnigen et al., 2013).

Strongly connected components in the edge-weighted islet-specific network were identified by ClusterONE, a non-partitioning graph decomposition algorithm (Nepusz et al., 2012), using a minimum density of 0.2, which is calculated as the average edge weight within the complex if missing edges are assumed to have a weight of zero, and a maximum overlap of 0.3 between two complexes before they were merged using the multi-merge option, and otherwise default parameters. ClusterONE uses the matching score as default for calculating the overlap between two complexes, which is defined as the intersection size squared, divided by the product of the sizes of the two complexes.

A three-step approach was applied to define spoke-hub-complexes. First, for each gene in the network a complex was defined by all its first order interaction partners. Next a topology filter was applied to prune complexes for interaction partners that tend to interact with many proteins in an unspecific way, due to either experimental artifacts or for biological reasons. In brief, genes were removed from the complex if <5% of its interaction partners were within the given complex. Lastly, overlapping clusters were merged using the same approach as for ClusterONE. Since this approach ignores edge-weights it was applied to the node-removal version of the islet-specific protein interaction network.

Finally, overlapping complexes resulting from the two approaches were merged using the same approach as before. Complexes with fewer than 6 or more than 50 nodes were discarded in the downstream analysis, resulting in 3,692 islet complexes. Diameter and average degree, clustering coefficient and betweenness-centrality were calculated for each complex using the igraph R-package (Csardi and Nepusz, 2006).

### Coordinated expression of protein complexes

The TissueRanker approach (Börnigen et al., 2013) utilizes the assumption that a mutation in a hub-spoke complex is likely to have an affect in tissues where the proteins within the complex show high degree of coordinated expression and thus, that the degree of coordinated expression may aid in prioritizing tissues in which the complex is active and where deregulation of the complex could be detrimental. Here we extended the methodology to complexes with more complex topology. In brief the $PCC.mean_{c}^{t}$ for complex *c* in tissue *t* is defined as the average pairwise Pearson correlation coefficient (*PCC*) of gene expression (RPKM values) between any two interacting genes within the complex for the given tissue:

$$\begin{array}{lll} PCC_{xy}^{t} & = & \frac{\sum_{i=1}^{N_{s}}\left(x_{i}^{t}-\bar{x^{t}}\right)\left(y_{i}^{t}-\bar{y^{t}}\right)}{\sqrt{\sum_{i=1}^{N_{s}}{\left(x_{i}^{t}-\bar{x^{t}}\right)}^{2}}\sqrt{\sum_{i=1}^{N_{s}}{\left(y_{i}^{t}-\bar{y^{t}}\right)}^{2}}}\\ PCC.mean_{c}^{t} & = & \frac{\sum_{x=1}^{N_{g}}\sum_{y\in I_{x}}PCC_{xy}^{t}}{2\;\cdot \;N_{e}} \end{array}$$

where *N*_*s*_ is the number of samples for tissue *t, N*_*g*_ is the number of genes in protein complex *c, N*_*e*_ is the number of edges in protein complex *c*, and *I*_*x*_ is the interaction partners of gene *x* excluding any self-loops.

To alleviate any potential bias arising from different numbers of tissues samples (Börnigen et al., 2013) we further standardized the $PCC.mean_{c}^{t}$ values within a tissue by first converting the average correlation coefficients to an approximately normal distribution using Fisher transformation:

$$\begin{array}{lll} z_{c}^{t} & = & \frac{1}{2}\ln\;\frac{1+PCC.mean_{c}^{t}}{1-PCC.mean_{c}^{t}}\\ CE_{c}^{t}=\;\frac{z_{c}^{t}-\mu^{t}}{\sigma^{t}} \end{array}$$

The resulting *z*-scores are here referred to as coordinated expression (CE) and used to compare tissue relevance across tissues for a given complex.

RPKM values from RNAseq data for 31 tissues from the Genotype-Tissue Expression (GTEx) project were obtained through the database of Genotypes and Phenotypes (dbGaP) (study accession phs000424.v4.p1, version from 17/1-2014; Mailman et al., 2007). However, since the GTEx data does not include pancreatic islets, RNAseq data for whole islets, beta cells, and non-beta cells (from pancreatic islets; Nica et al., 2013) were combined with the GTEx data.

We defined 1,007 islet complexes with coordinated expression as the subset of the 3,692 islet complexes where at least one of the islet tissue components (whole islet, beta, and non-beta cells) was among the three tissues with highest coordinated expression level among the 34 included tissues.

### Compiling islet biology and islet diabetic phenotype related gene sets

We compiled a set of 13 complementary sets of genes associated with T2D, monogenic forms of diabetes and related metabolic phenotypes (Table 1). These 13 gene sets are collectively referred to as islet diabetic phenotype gene sets and were chosen because of their relevance to the islet tissue.

We obtained GWAS SNPs and genes supported by gene-based tests for T2D (Morris et al., 2012; Mahajan et al., 2014), fasting glucose (Dupuis et al., 2010; Scott et al., 2012), 2 hour glucose (2 h glu) during an oral-glucose tolerance test (Dupuis et al., 2010; Scott et al., 2012), and proinsulin (Strawbridge et al., 2011). SNPs in GWAS loci were mapped to a gene if they fell within 110 kb upstream or 40 kb downstream of its transcription start and stop sites respectively, as these boundaries have been shown to capture the majority of *cis*-eQTLs associations (Veyrieras et al., 2008; Ardlie et al., 2015). We additionally included all genes that were reported in eQTL associations for the GWAS SNPs from the respective publications (Dupuis et al., 2010; Voight et al., 2010; Strawbridge et al., 2011; Morris et al., 2012; Scott et al., 2012; Mahajan et al., 2014). We also included genes harboring rare variants associated with either fasting glucose and T2D (Albrechtsen et al., 2013; Flannick et al., 2014; Steinthorsdottir et al., 2014; Wessel et al., 2015) or insulin processing and secretion (Huyghe et al., 2013). Genes associated with monogenic forms of diabetes were obtained from a literature review (McCarthy, 2010) and a curated list from a previous study (Morris et al., 2012).

Genes differentially expressed in islets were obtained from a study by Taneera et al. (2012). In addition, two other microarray datasets of beta-cell and islet gene expression, respectively, were obtained from the Gene Expression Omnibus database (accession IDs: GSE20966 and GSE25724) and differential gene expression between T2D patients and non-diabetic controls evaluated using the “limma” R package as implemented in the NCBI GEO2R tool. Genes with *P* < 0.001 were included in the gene sets, except for the dataset by Dominguez et al. (2011) where a stricter cutoff of *P* < 0.0001 was applied due to inflated significance values. We further included additional gene sets defined by the islet gene expression study from Taneera et al. (2012), namely genes that showed *cis-* or *trans*-eQTL associations with T2D associated SNPs and genes that were co-expressed with >2 T2D candidate genes. Finally, we included genes that were differentially methylated in islets in T2D patients compared to non-diabetic controls (Volkmar et al., 2012) or were both differentially methylated and differentially expressed (Dayeh et al., 2014).

We furthermore constructed four gene sets related to islet function, referred to as islet biology gene sets (Table 1). These sets included genes with islet-specific expression (Morán et al., 2012), genes in islet-selective open chromatin regions or genes overlapping clusters of islet-selective open chromatin sites (Gaulton et al., 2010) and genes manually curated as islet important (Pasquali et al., 2014).

Finally, we obtained at list of proteins that are targets of FDA approved drugs from the druggable human proteome (Uhlén et al., 2015).

The direct overlap of the gene sets was tested using a hypergeometric test with all 22,766 human genes as background.

### Functional convergence testing

To test the protein complexes for potential dysregulation in T2D, the degree of functional convergence of diabetes related genes was assessed. For each complex, the enrichment of each of the 13 islet diabetic phenotype gene sets was first calculated using a hypergeometric test and the corresponding *P*-values were next combined using Fisher's combined probability test (*P*_*combined*_).

The likelihood of observing a similar degree of functional convergence by chance was estimated for each complex by randomly sampling 100,000 sets of the same number of genes from the whole network. An empirical *P*-value (*P*_*emp*_) was then calculated by counting how many of these 100,000 random sets had a *P*_*combined*_ ≤ the real case divided by the number of random sets (*n* = 100,000). *P*_*emp*_ was adjusted for multiple hypotheses testing across complexes using a Benjamini–Hochberg correction and complexes with *P*_*emp*.*adj*_ < 0.05 were considered significant and thus showing potential T2D dysregulation. Genes in the gene sets without any interaction partners were excluded from the test.

### Functional annotation of protein complexes

We downloaded 3,906 biological pathways from ConsensusPathDB release 30 (Kamburov et al., 2013). Over-representation analysis of pathways was tested using a hypergeometric test. In short, all gene sets with at least two candidate genes were tested. The background was restricted to the subset of all genes within the protein interaction network that participate in at least one pathway and similarly, only input genes that were part of the background were included for testing.

### Testing for enrichment of diabetes-related GWAS signal

We further investigated whether the complexes with potential T2D dysregulation were enriched for association with T2D or glycemic traits in 19 different GWA-studies (Supplementary Table 6) using the MAGENTA method (Segrè et al., 2010).

The analysis was repeated using three definitions of complexes: (1) including all genes in the complexes, (2) excluding genes from the complex that had genome-wide significant *P*-values (*P* < 5 × 10^−8^) in the respective GWAS, i.e., different genes are excluded from the complexes when testing enrichment in the different GWAS studies, and (3) excluding genes that were used for input in the corresponding gene set. For example, all fasting glucose associated genes were excluded from the complexes when testing for enrichment using “Fasting glucose” and “Fasting glucose, Manning” but not when testing for enrichment of e.g., “Fasting insulin”.

In the MAGENTA analysis we used the 95^th^ percentile of all gene *P*-values as the *P*^*cutoff*^ and SNPs in GWAS loci were mapped to a gene if they fell within 110 kb upstream or 40 kb downstream of its transcription start and stop sites, respectively.

### Statistical analysis and visualization

Statistical analyses were performed in the statistical computing language R (R Core Team, 2014) and network visualizations were made in R using the igraph package (Csardi and Nepusz, 2006). Tissue depictions in figures were adapted from Stumvoll et al. (2010).

## Author contributions

HP, VG, and SB conceived the study and provided the initial design and data analysis framework. HP and VG performed the analysis and drafted the original manuscript. HP, VG, and SB contributed to the interpretation and corresponding text. All authors approved the version to be published.

## Funding

The Technical University of Denmark has received support from the Innovative Medicines Initiative Joint Undertaking under grant agreement no. 115317 (DIRECT), resources of which are composed of financial contribution from the European Union's Seventh Framework Programme (FP7/2007–2013) and EFPIA companies in kind contribution. The Novo Nordisk Foundation Center for Protein Research, University of Copenhagen, is supported financially by the Novo Nordisk Foundation (Grant agreement NNF14CC0001).

### Conflict of interest statement

The authors declare that the research was conducted in the absence of any commercial or financial relationships that could be construed as a potential conflict of interest.

## Supplementary material

### Data availability

The 24 complexes with potential T2D dysregulation are provided in supplementary Data Sheet 2 and further visualized in Data Sheet 1.
