# Supplementary material for: Pancreatic Islet Protein Complexes and Their Dysregulation in Type 2 Diabetes
Source: Front Genet. 2017 Apr 20;8:43. doi: 10.3389/fgene.2017.00043 (PMC5397424; doi:10.3389/fgene.2017.00043)
Supplement: Supplementary file 2 [file DataSheet1.pdf]

Islet diabetic phenotype gene sets

Color legend:

- T2D expression (C)
- T2D expression (B)
- Glycemic gene−based
- Co−expression
- T2D eQTL
- T2D expression (A)
- Hyperglycemia expression
- T2D methylation (B)
- T2D methylation (A)
- Monogenic
- OMIM
- T2D GWAS/rare variant
- Glycemic GWAS/rare variant

Islet biology gene sets

Color legend:

- Islet specific
- Open chromatin
- Open chromatin clusters
- Islet biology

Visualization of the 24 complexes with potential T2D dysregulation, where the evidence sources supporting the different genes are annotated.

The first page (of the 25 pages) in this file shows the color legend for the diabetic phenotype and islet related gene sets. Nodes with red boarder indicate targets of FDA approved drugs.

Complex−1

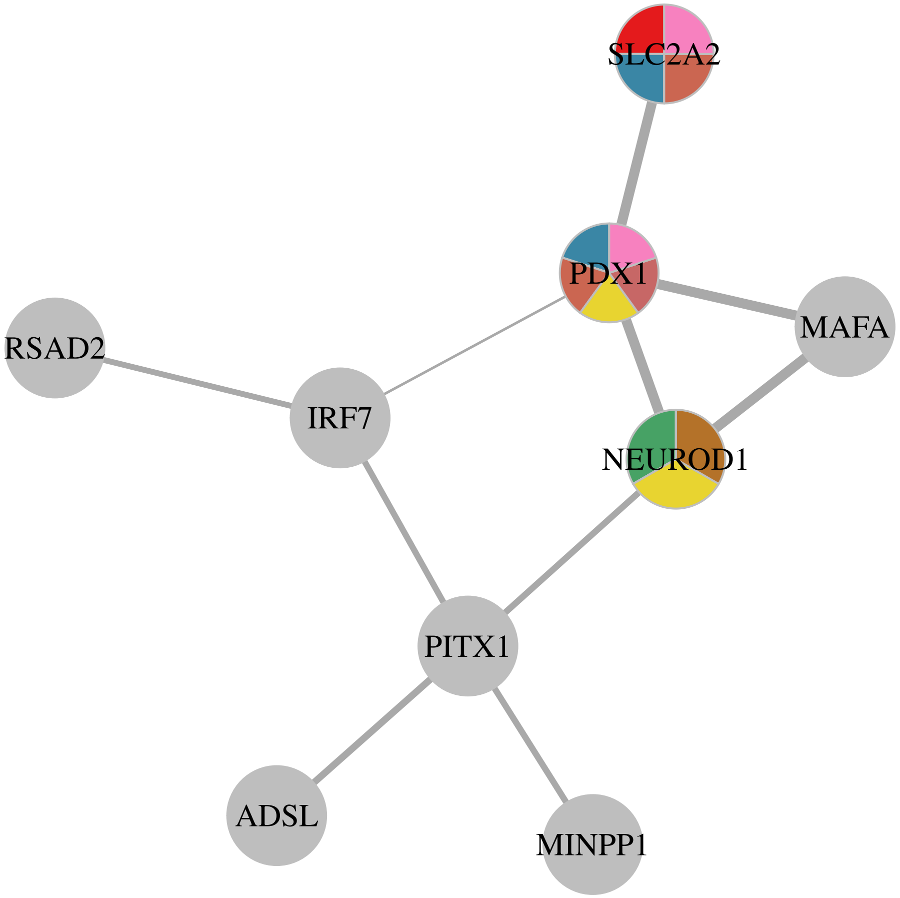

Emperical P−value: < 1E−5
Number of genes in complex: 9
Top coordinated expressed tissues: Colon, Skin, Bone Marrow

Node color based on: Islet diabetic phenotype gene sets

Complex−1

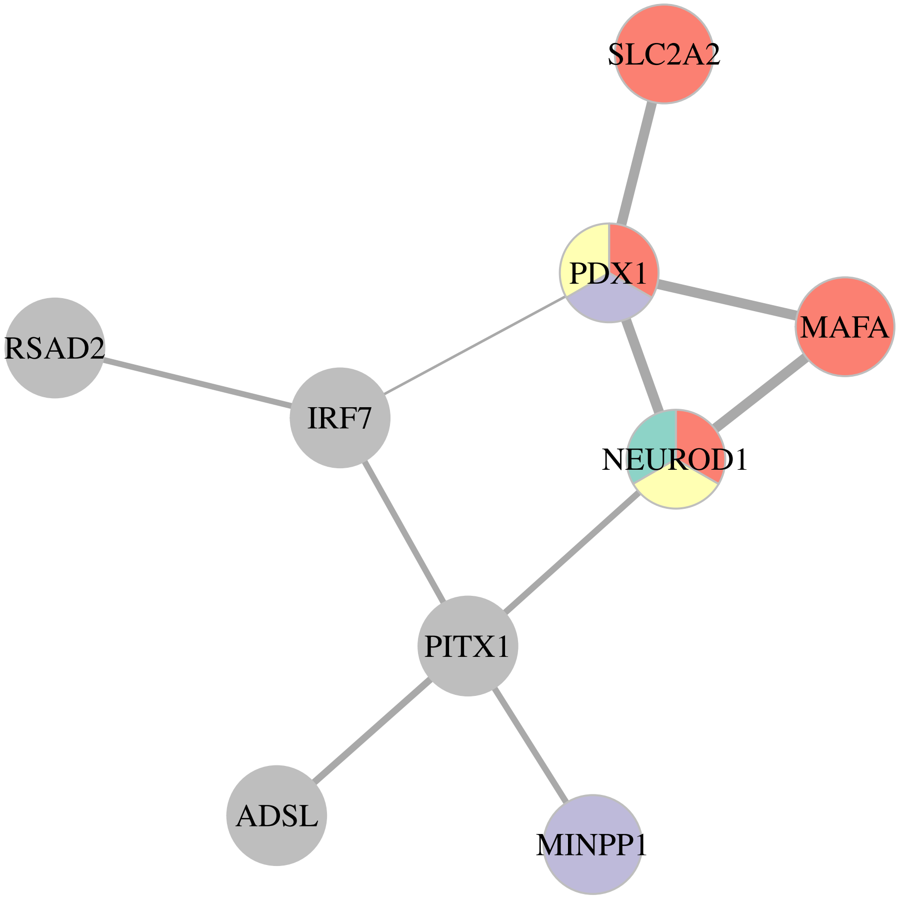

Emperical P−value: < 1E−5
Number of genes in complex: 9
Top coordinated expressed tissues: Colon, Skin, Bone Marrow

Node color based on: Islet biology gene sets

Complex–2

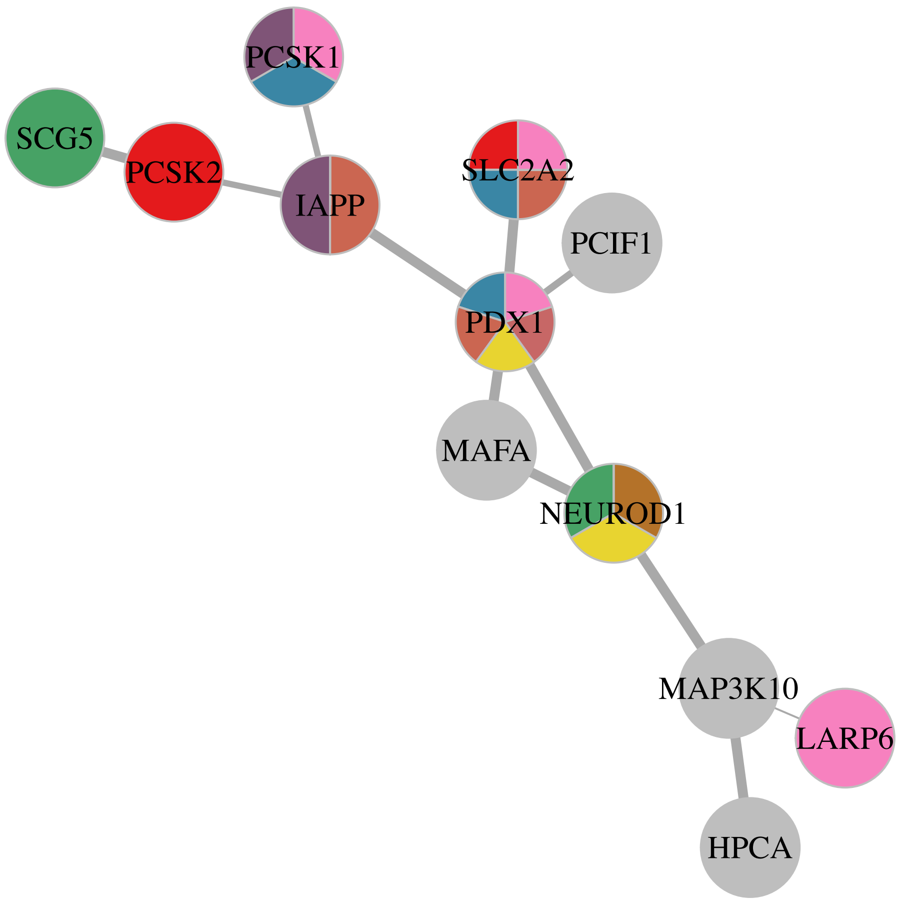

Emperical P–value: < 1E–5
Number of genes in complex: 12
Top coordinated expressed tissues: islet, Pituitary, Pancreas

Node color based on: Islet diabetic phenotype gene sets

Complex–2

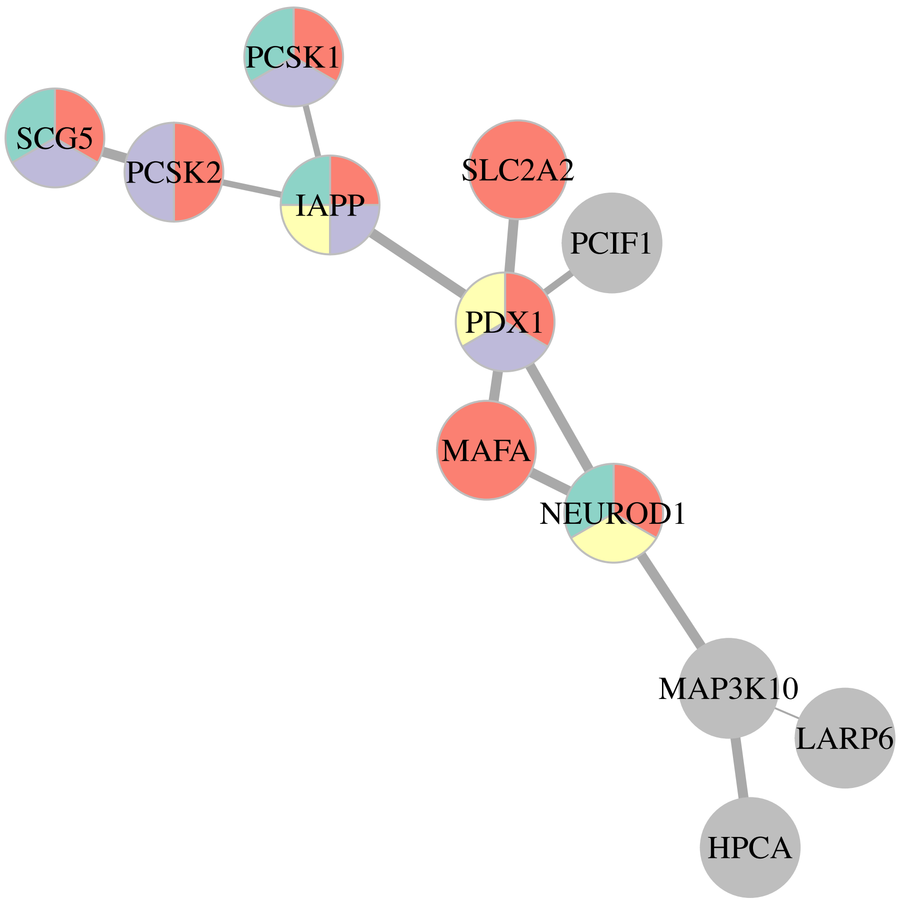

Emperical P–value: < 1E–5
Number of genes in complex: 12
Top coordinated expressed tissues: islet, Pituitary, Pancreas

Node color based on: Islet biology gene sets

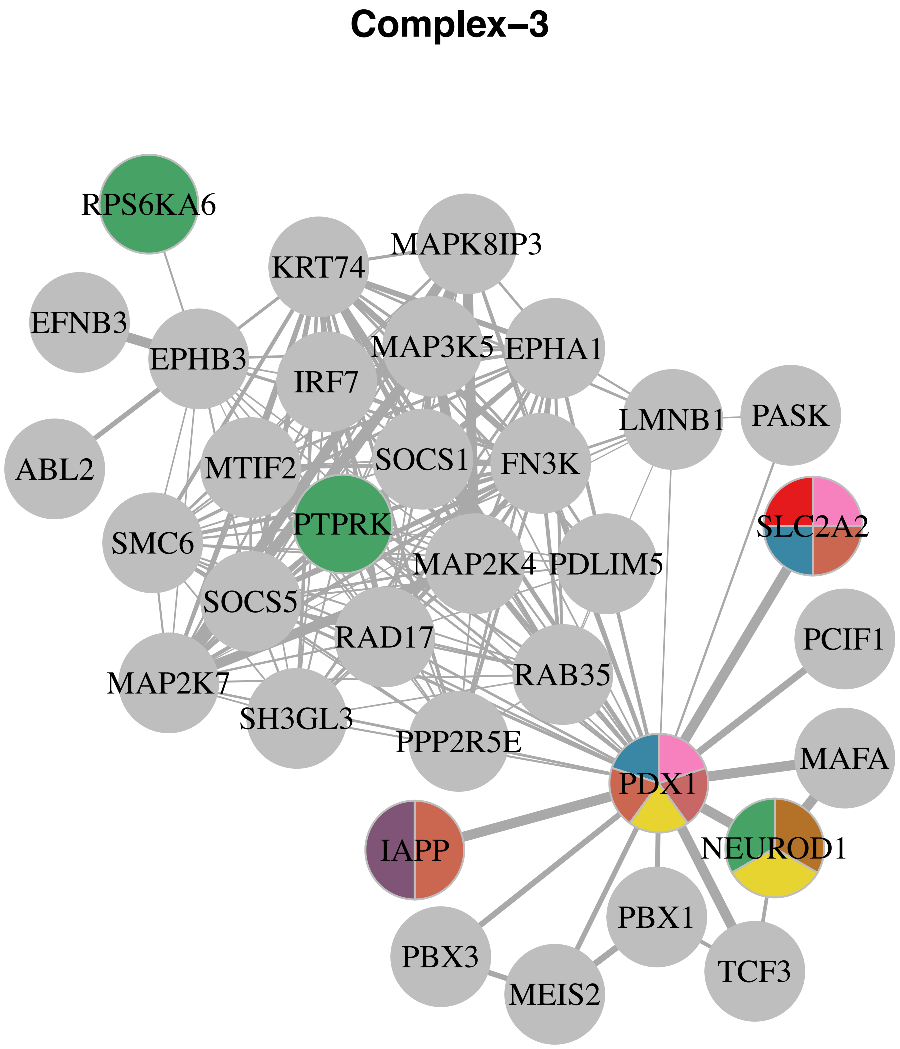

Emperical P–value: 2e–05
Number of genes in complex: 34
Top coordinated expressed tissues: Bladder, Ovary, Breast

Node color based on: Islet diabetic phenotype gene sets

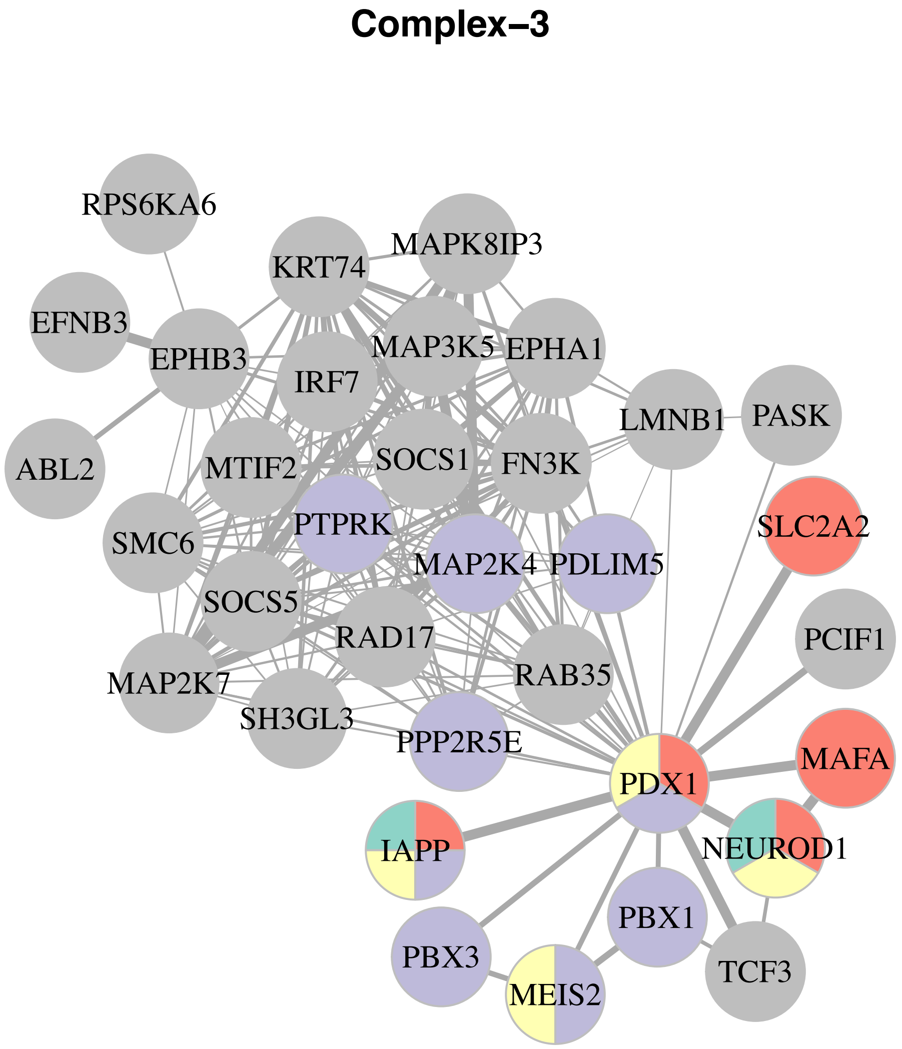

Emperical P–value: 2e–05
Number of genes in complex: 34
Top coordinated expressed tissues: Bladder, Ovary, Breast

Node color based on: Islet biology gene sets

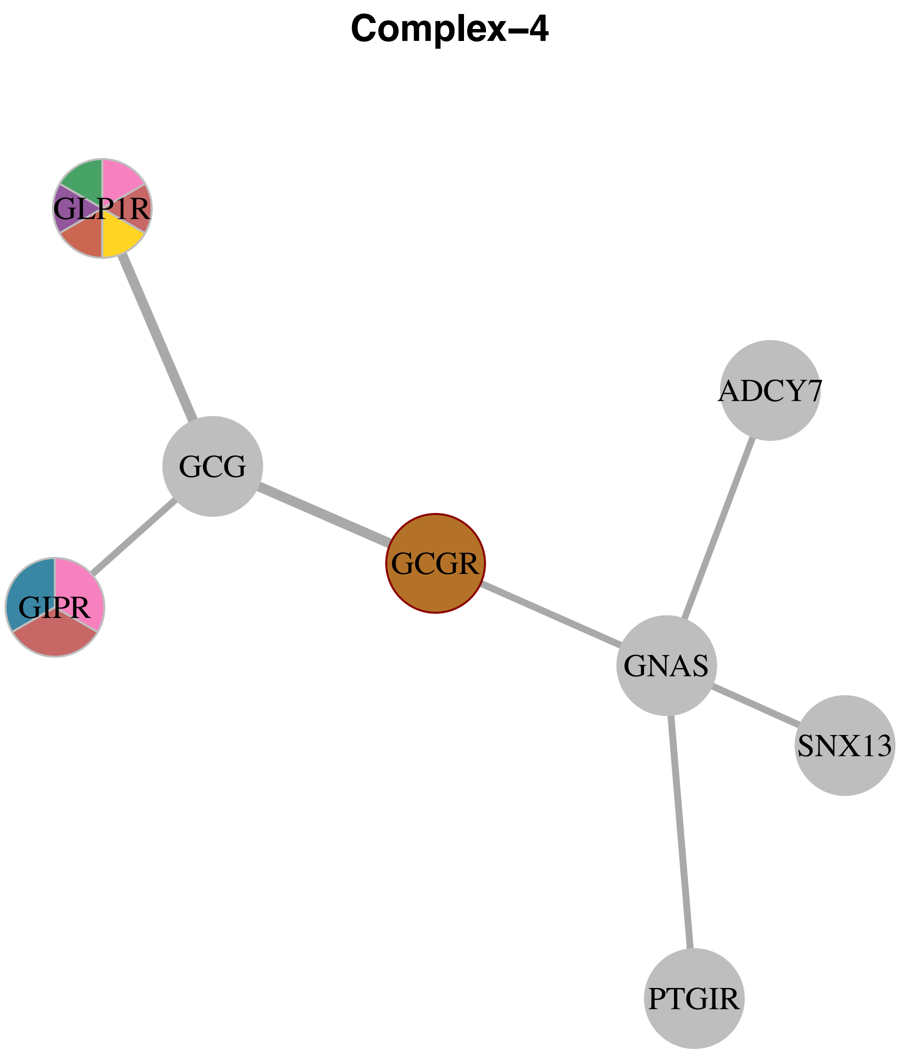

Emperical P−value: 3e−05
Number of genes in complex: 8
Top coordinated expressed tissues: islet, Pancreas, Small Intestine

Node color based on: Islet diabetic phenotype gene sets

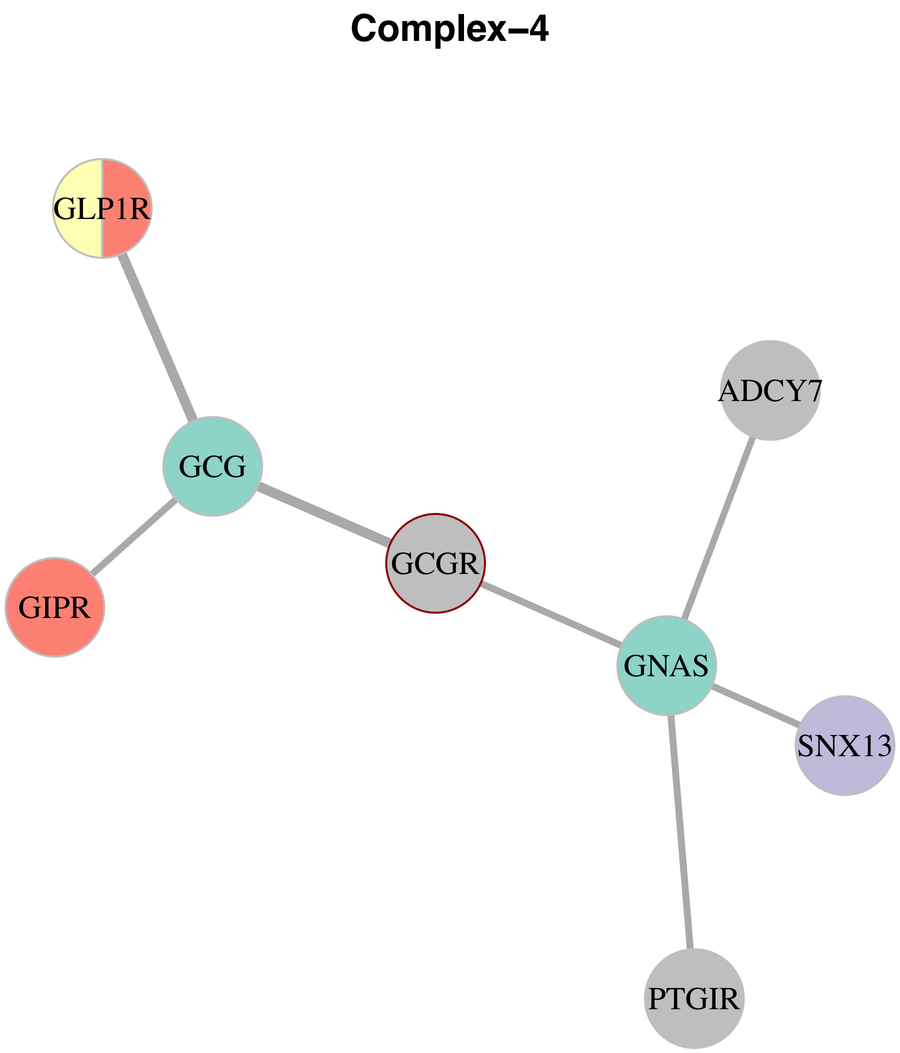

Emperical P−value: 3e−05
Number of genes in complex: 8
Top coordinated expressed tissues: islet, Pancreas, Small Intestine

Node color based on: Islet biology gene sets

Complex–5

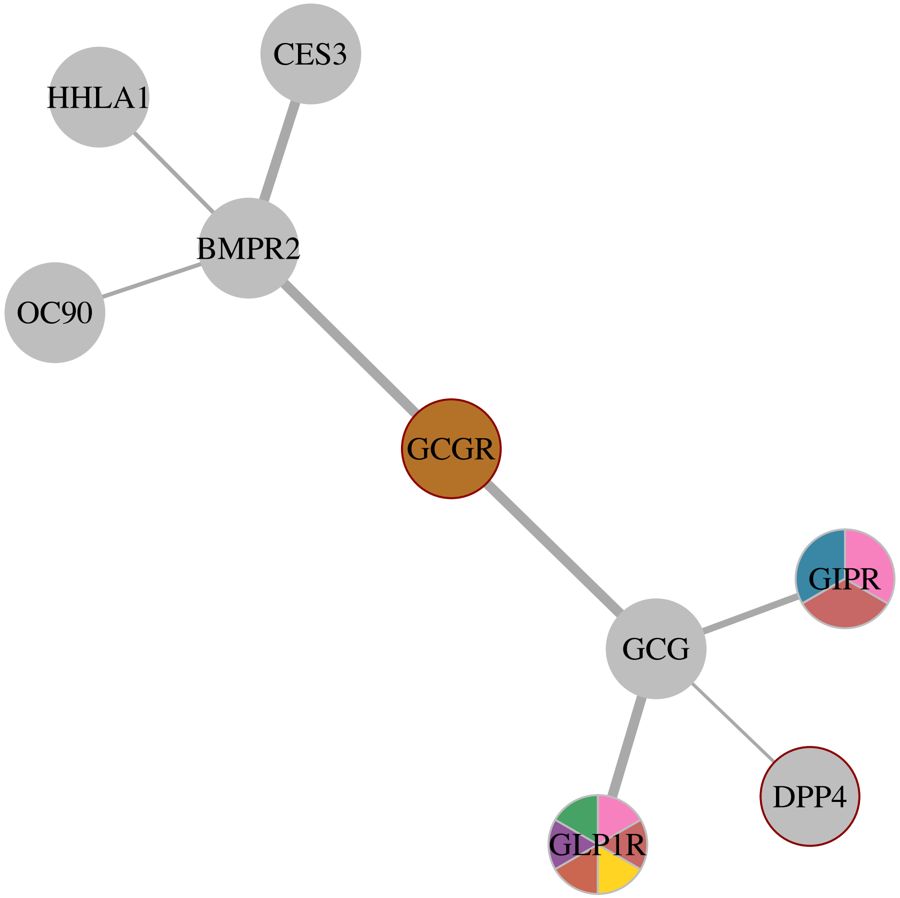

Emperical P–value: 3e–05
Number of genes in complex: 9
Top coordinated expressed tissues: islet, beta, Bone Marrow

Node color based on: Islet diabetic phenotype gene sets

Complex–5

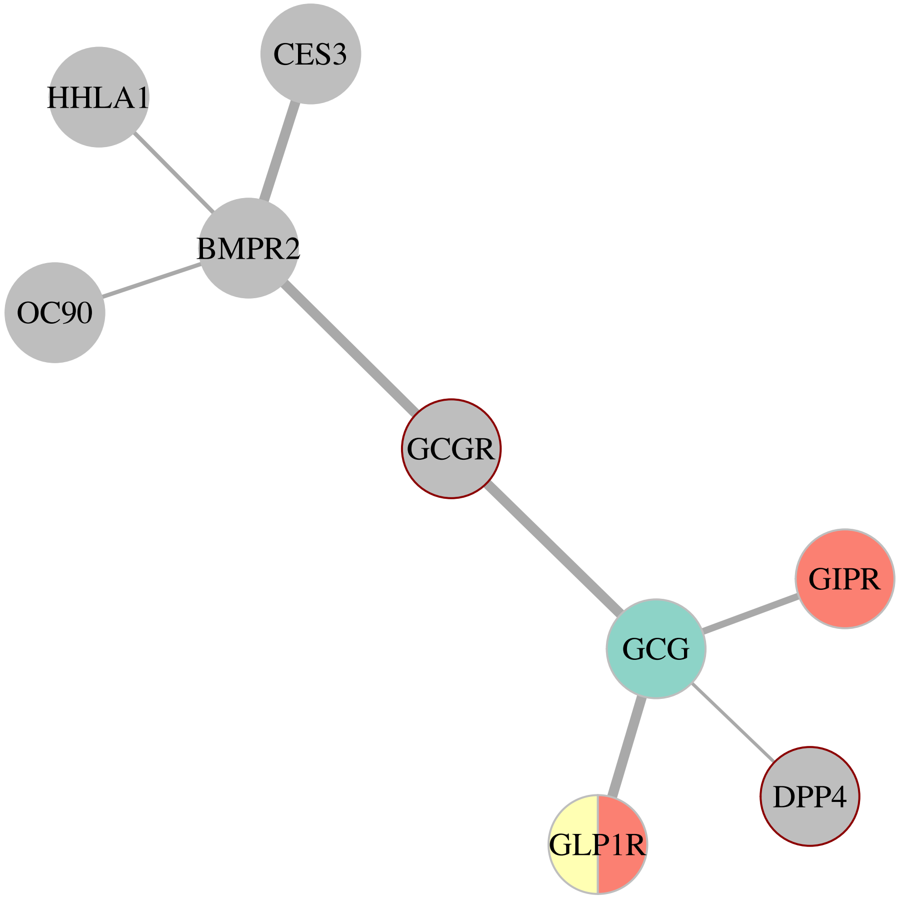

Emperical P–value: 3e–05
Number of genes in complex: 9
Top coordinated expressed tissues: islet, beta, Bone Marrow

Node color based on: Islet biology gene sets

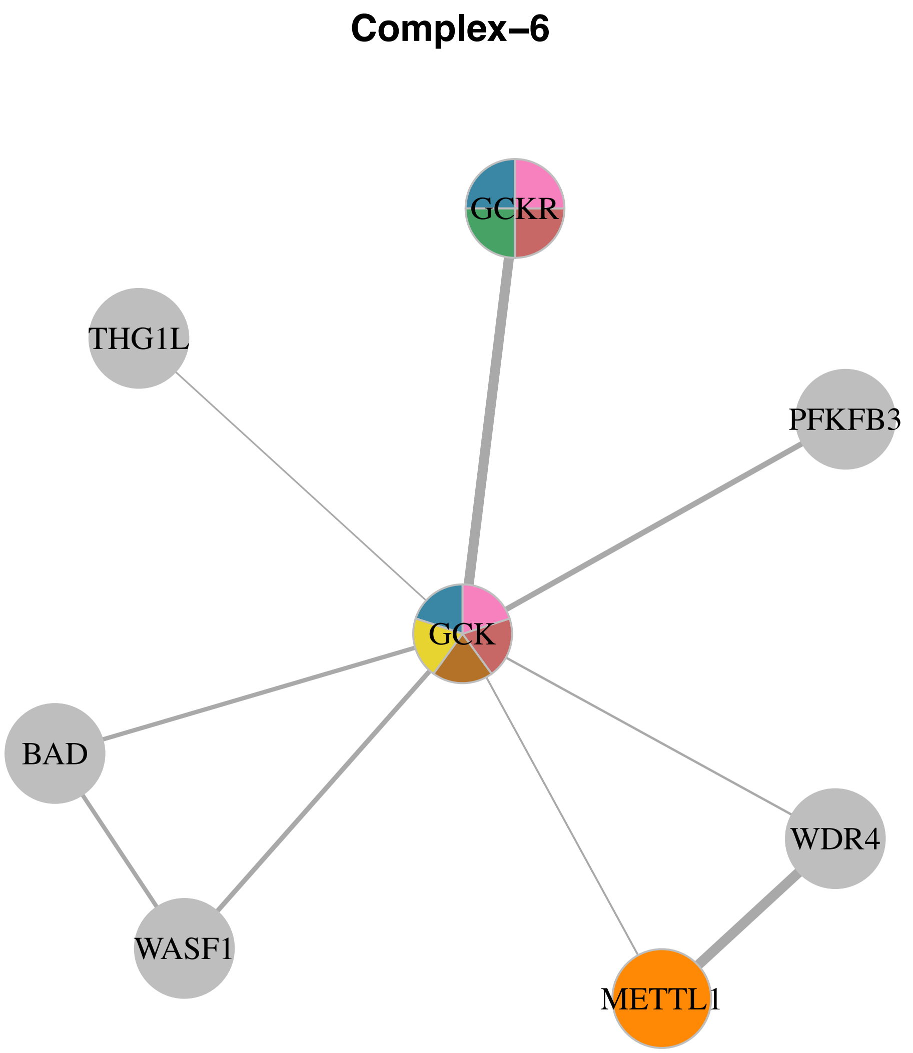

Emperical P–value: 3e–05
Number of genes in complex: 8
Top coordinated expressed tissues: Nerve, Cervix Uteri, Small Intestine

Node color based on: Islet diabetic phenotype gene sets

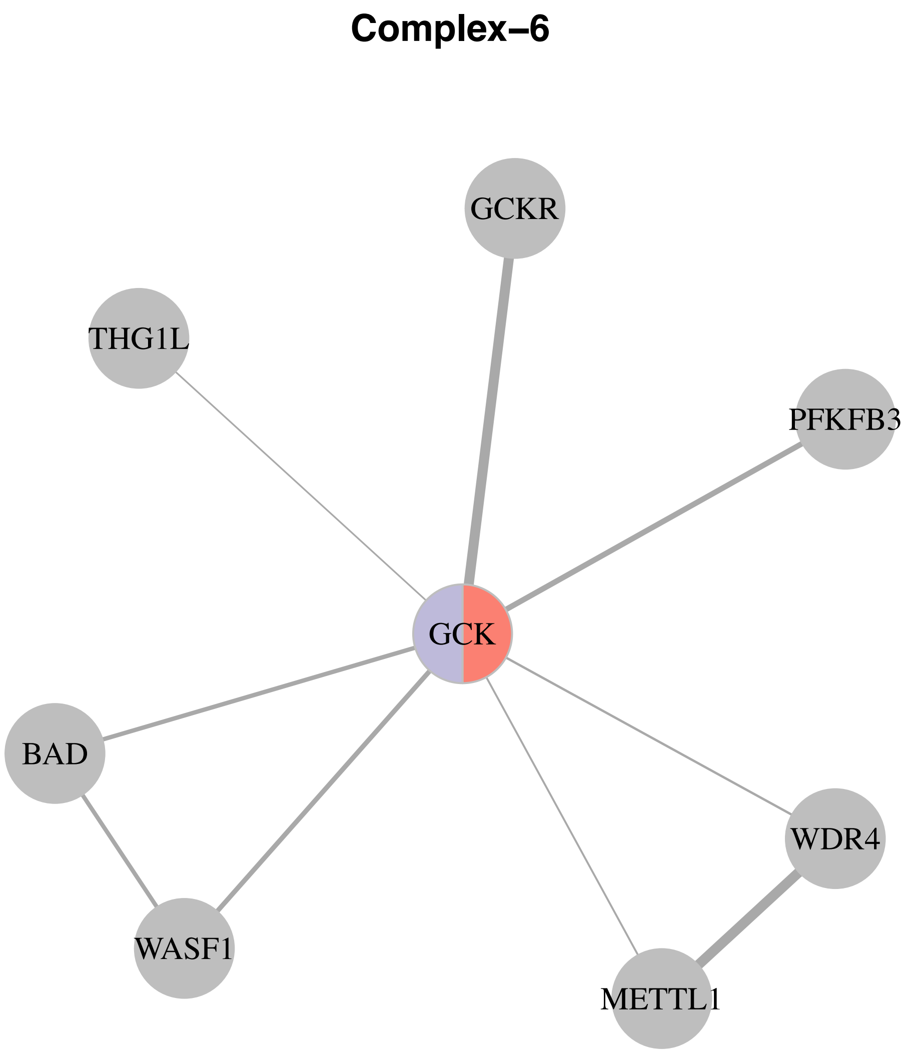

Emperical P–value: 3e–05
Number of genes in complex: 8
Top coordinated expressed tissues: Nerve, Cervix Uteri, Small Intestine

Node color based on: Islet biology gene sets

Complex–7

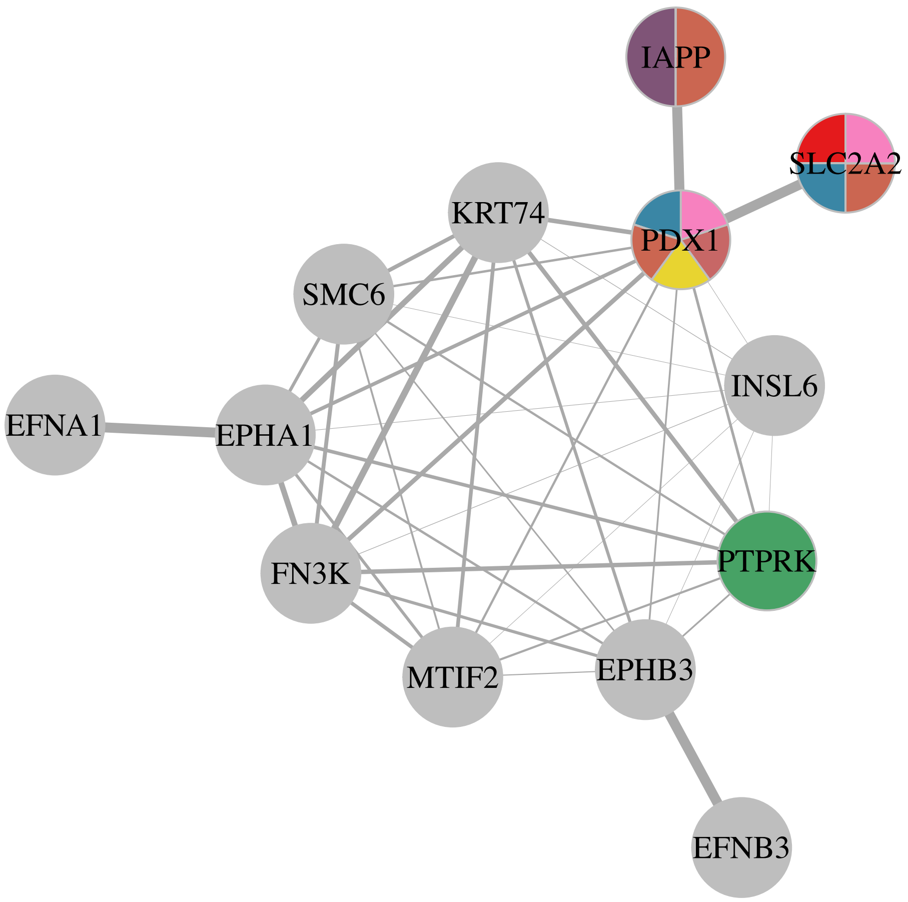

Emperical P–value: 4e–05
Number of genes in complex: 13
Top coordinated expressed tissues: Colon, Bladder, nonbeta

Node color based on: Islet diabetic phenotype gene sets

Complex–7

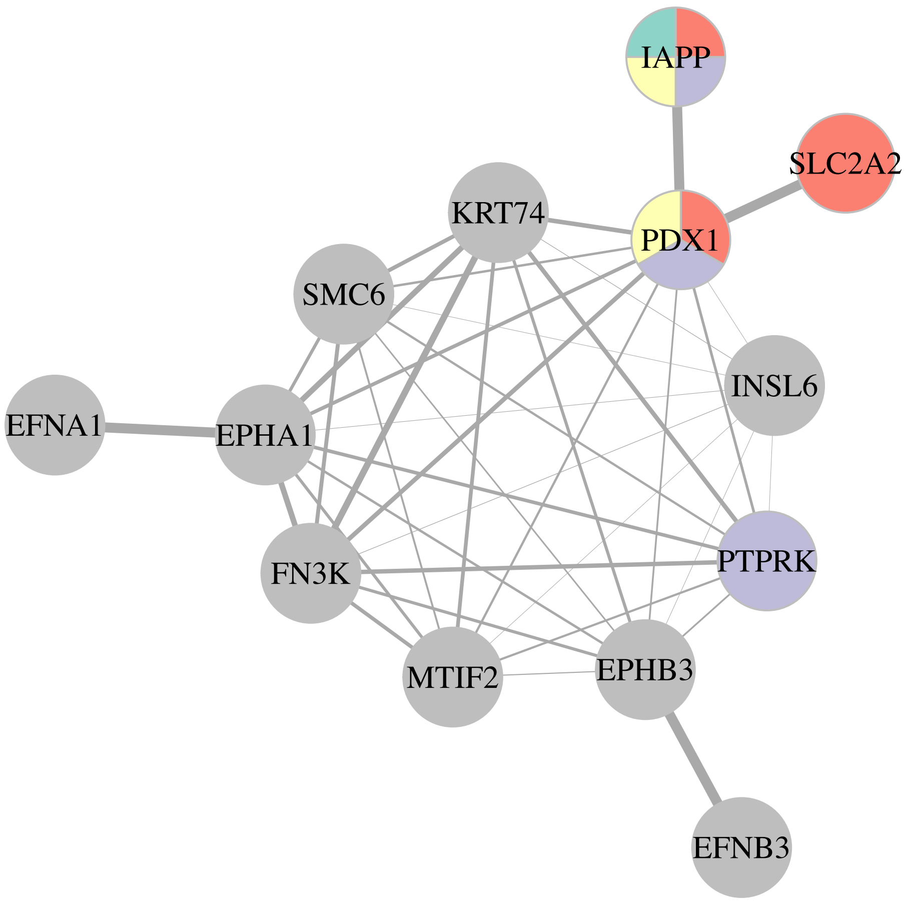

Emperical P–value: 4e–05
Number of genes in complex: 13
Top coordinated expressed tissues: Colon, Bladder, nonbeta

Node color based on: Islet biology gene sets

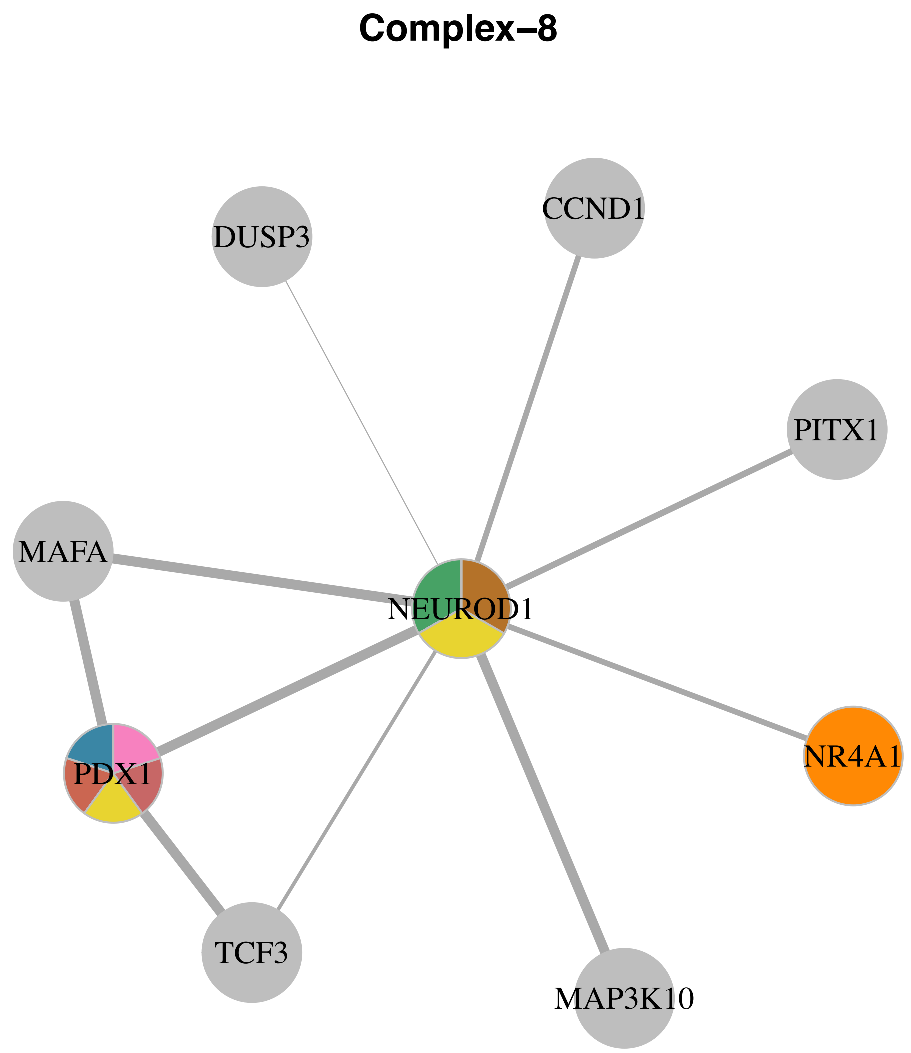

Emperical P–value: 4e–05
Number of genes in complex: 9
Top coordinated expressed tissues: Skin, Colon, Adrenal Gland

Node color based on: Islet diabetic phenotype gene sets

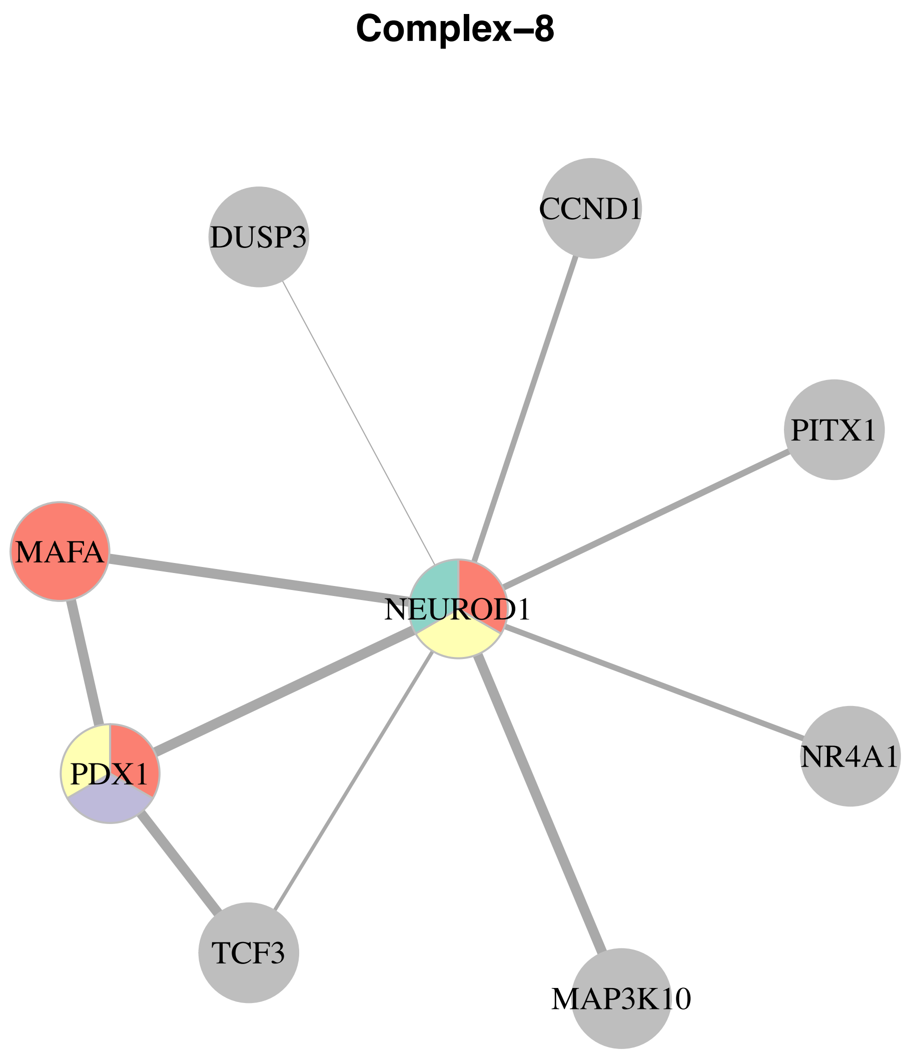

Emperical P–value: 4e–05
Number of genes in complex: 9
Top coordinated expressed tissues: Skin, Colon, Adrenal Gland

Node color based on: Islet biology gene sets

Complex–9

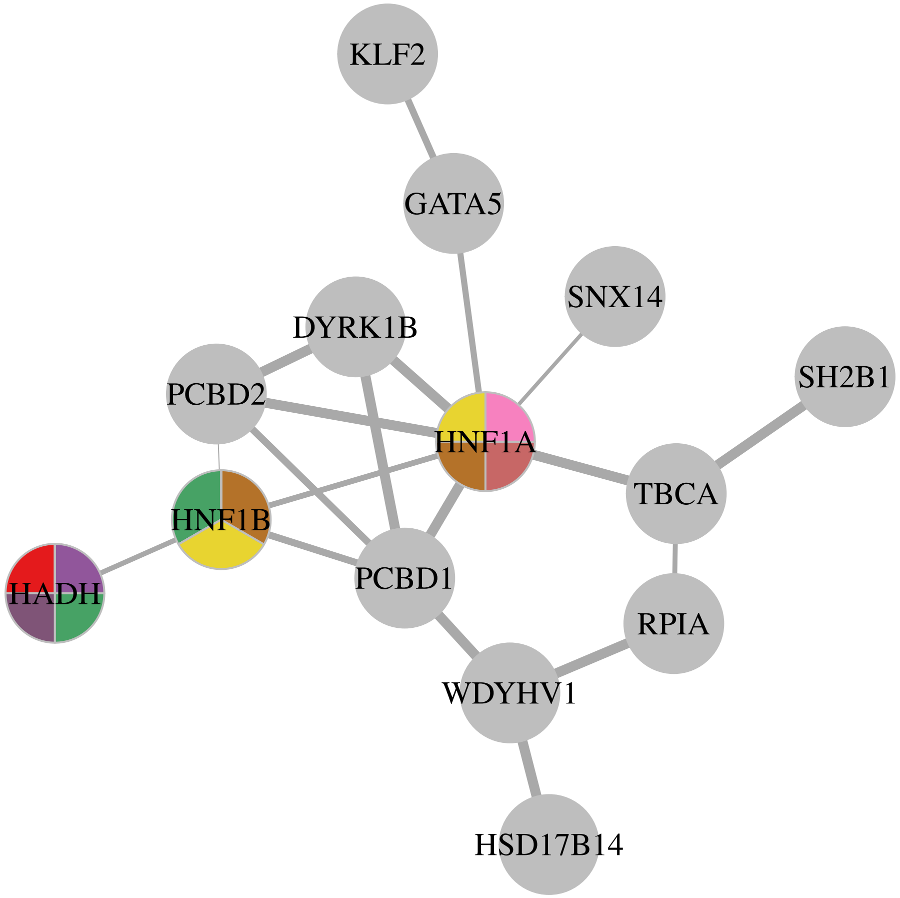

Emperical P–value: 5e–05
Number of genes in complex: 14
Top coordinated expressed tissues: Fallopian Tube, Uterus, Bladder

Node color based on: Islet diabetic phenotype gene sets

Complex–9

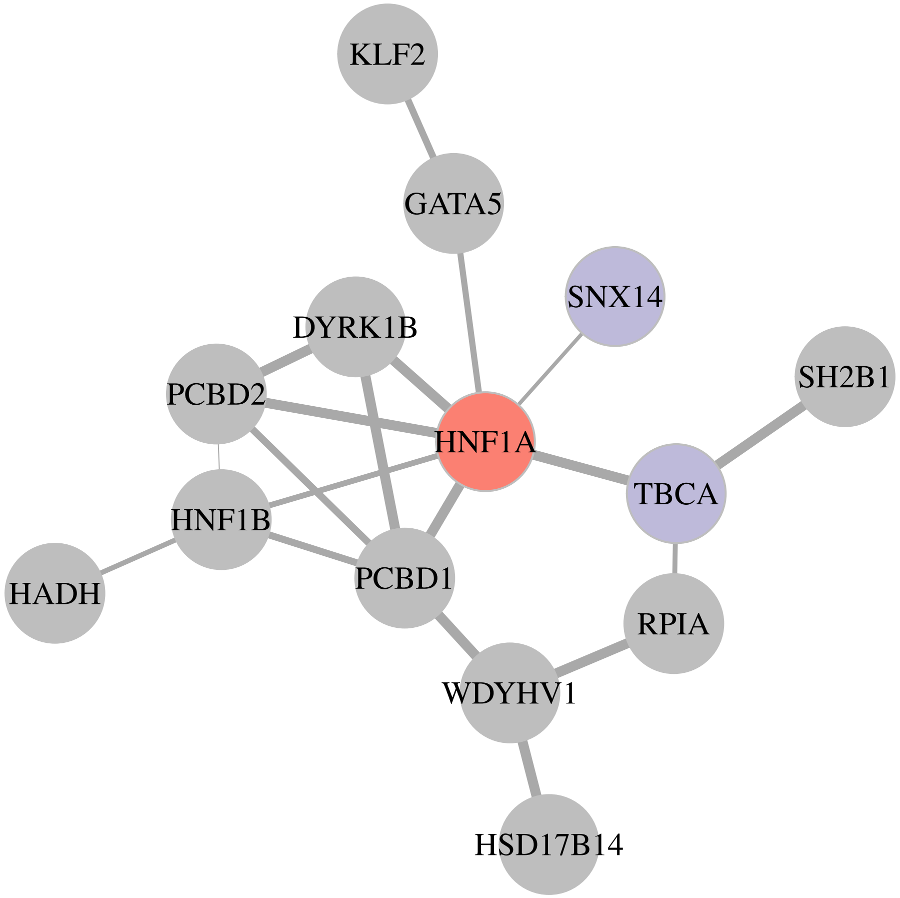

Emperical P–value: 5e–05
Number of genes in complex: 14
Top coordinated expressed tissues: Fallopian Tube, Uterus, Bladder

Node color based on: Islet biology gene sets

Complex−10

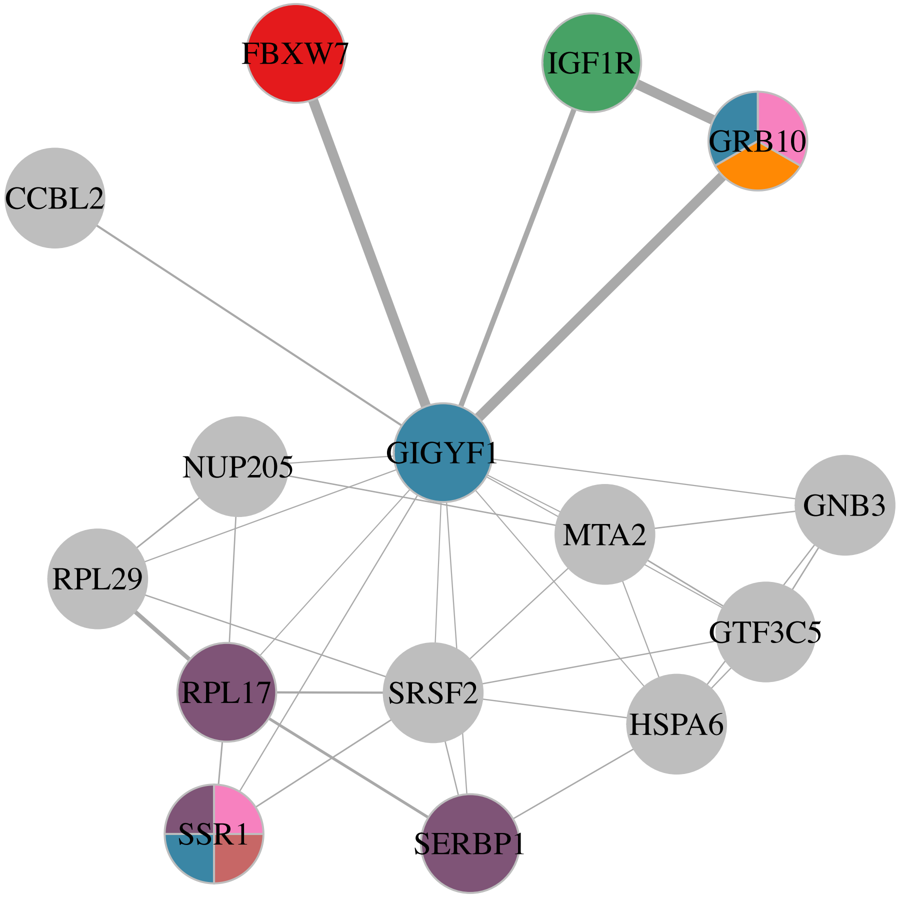

Emperical P−value: 6e−05
Number of genes in complex: 15
Top coordinated expressed tissues: Small Intestine, Liver, Brain

Node color based on: Islet diabetic phenotype gene sets

Complex−10

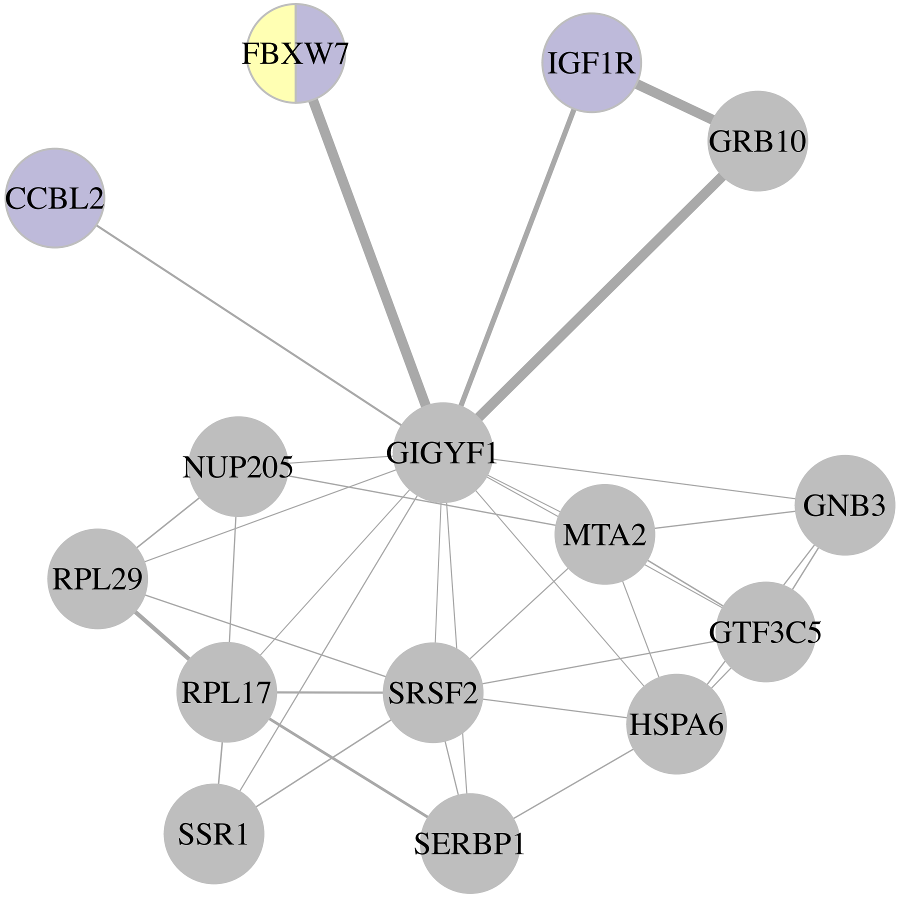

Emperical P−value: 6e−05
Number of genes in complex: 15
Top coordinated expressed tissues: Small Intestine, Liver, Brain

Node color based on: Islet biology gene sets

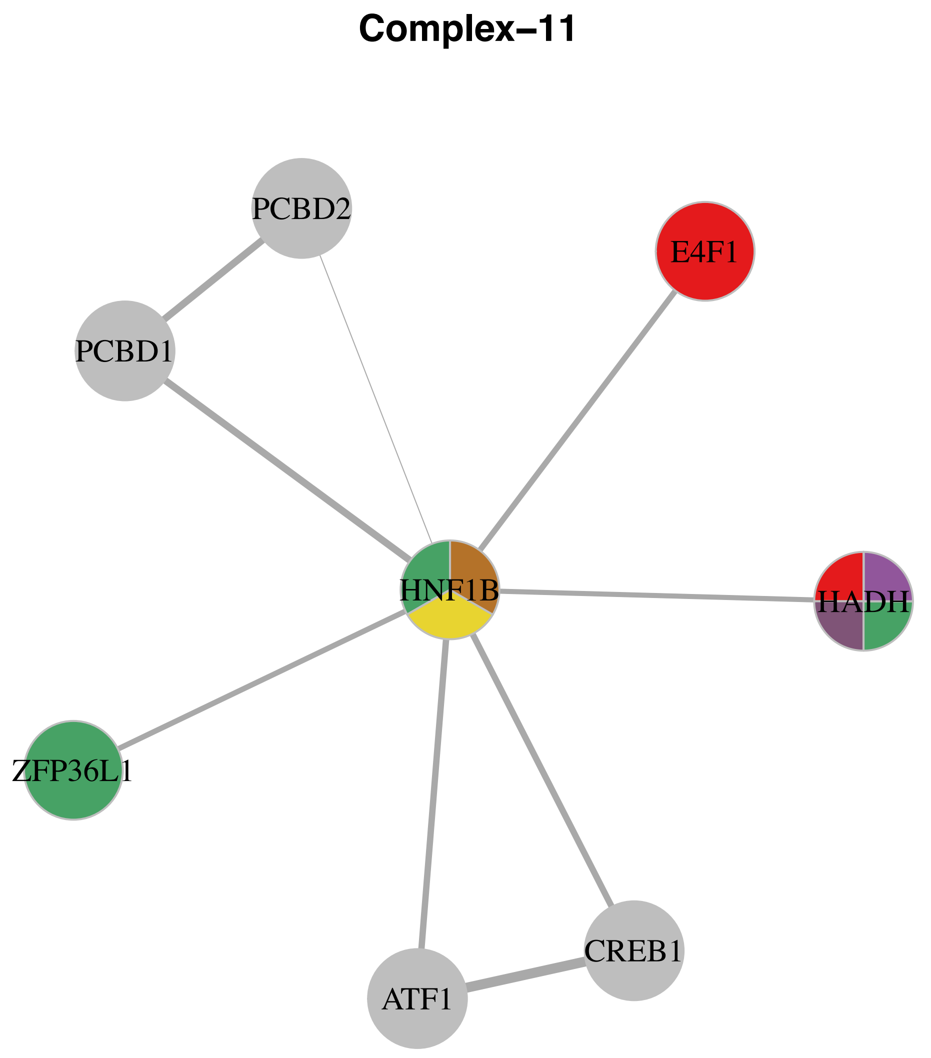

Emperical P−value: 6e−05
Number of genes in complex: 8
Top coordinated expressed tissues: Bladder, Fallopian Tube, Prostate

Node color based on: Islet diabetic phenotype gene sets

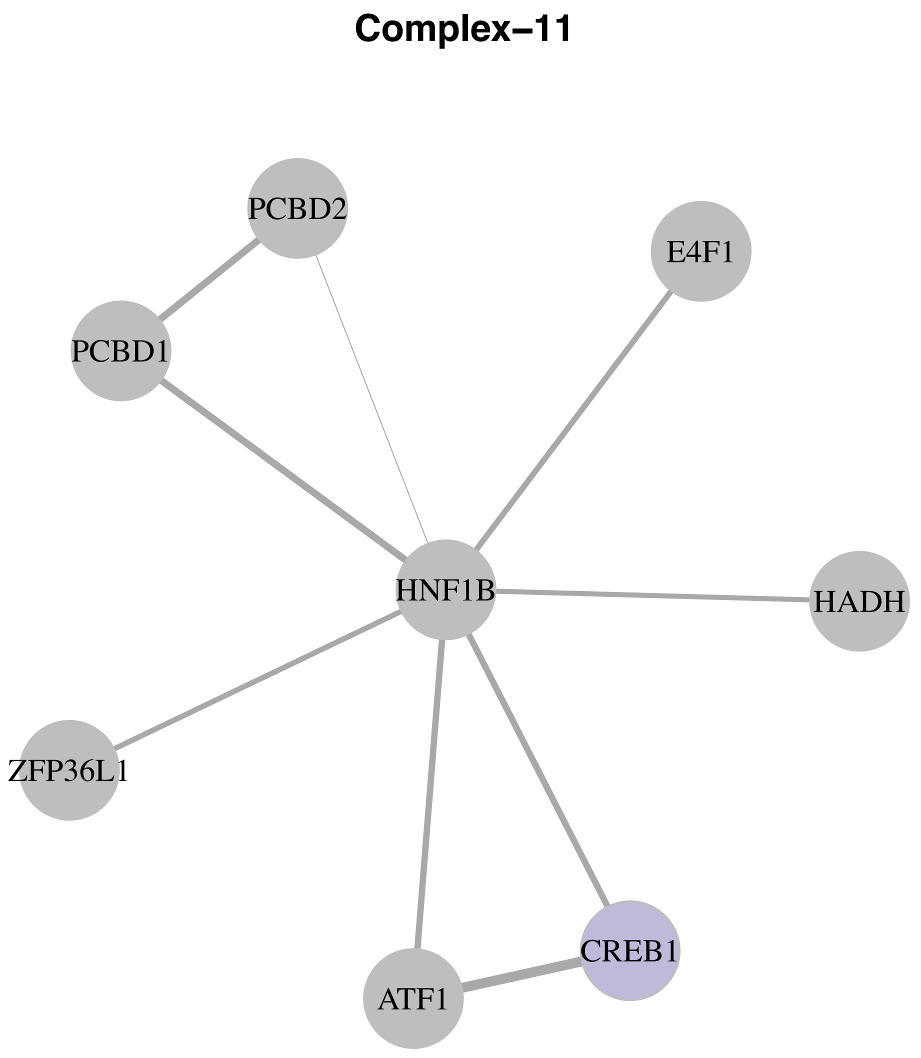

Emperical P−value: 6e−05
Number of genes in complex: 8
Top coordinated expressed tissues: Bladder, Fallopian Tube, Prostate

Node color based on: Islet biology gene sets

Complex−12

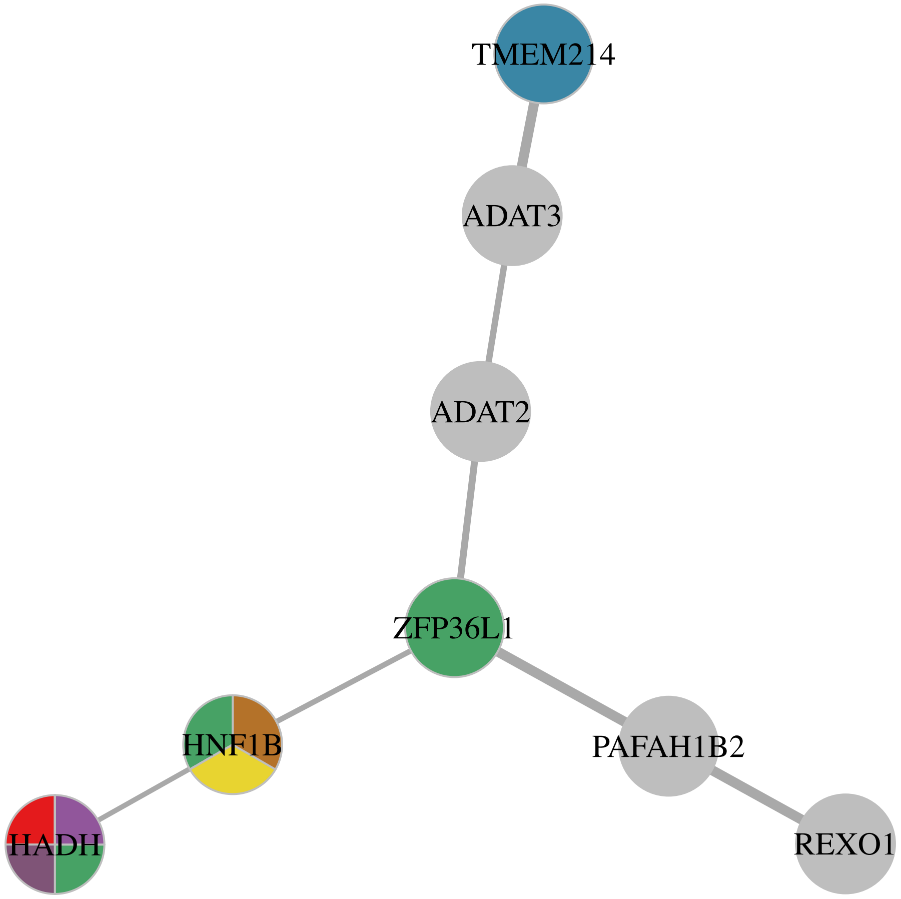

Emperical P−value: 6e−05
Number of genes in complex: 8
Top coordinated expressed tissues: Bladder, Fallopian Tube, Cervix Uteri

Node color based on: Islet diabetic phenotype gene sets

Complex−12

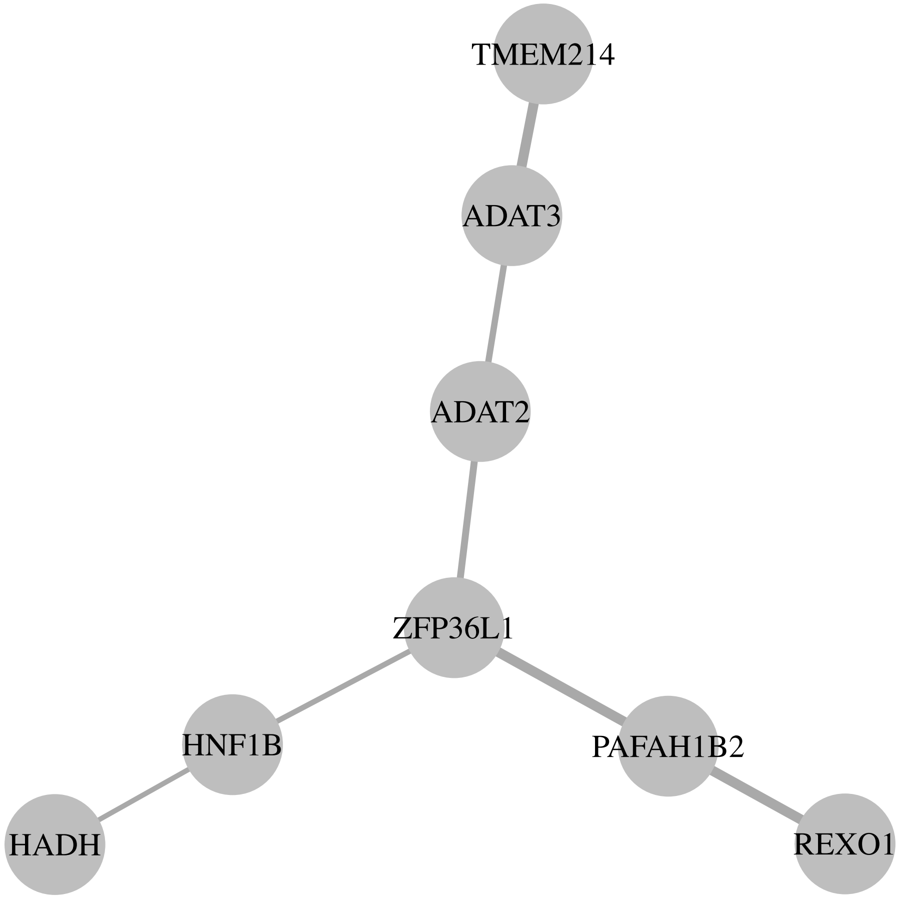

Emperical P−value: 6e−05
Number of genes in complex: 8
Top coordinated expressed tissues: Bladder, Fallopian Tube, Cervix Uteri

Node color based on: Islet biology gene sets

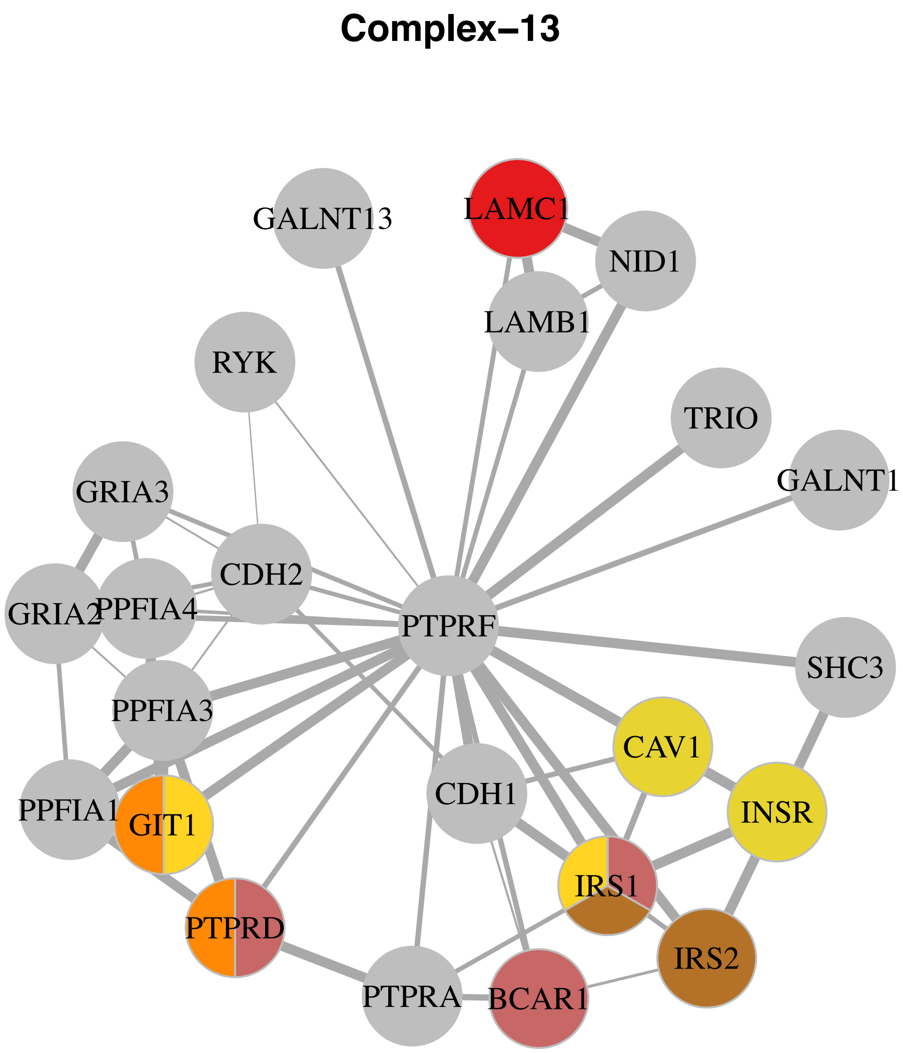

Emperical P−value: 7e−05
Number of genes in complex: 24
Top coordinated expressed tissues: beta, islet, Testis

Node color based on: Islet diabetic phenotype gene sets

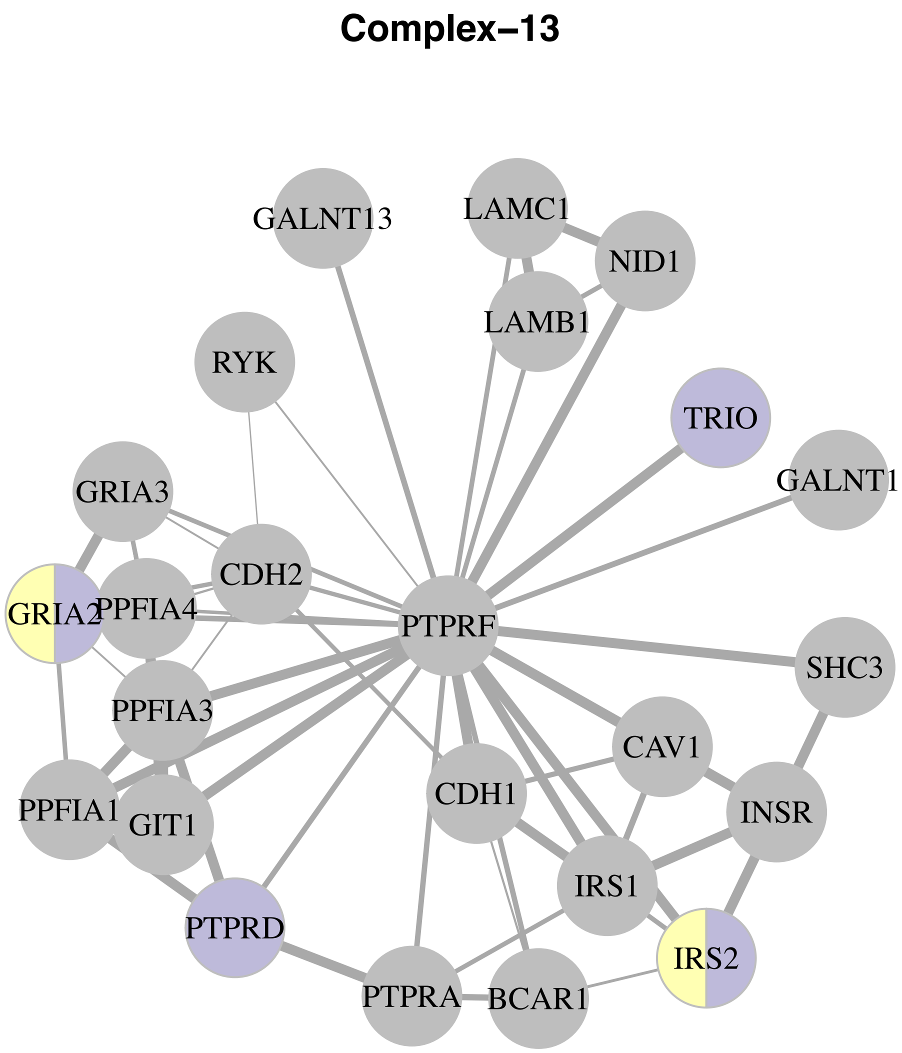

Emperical P−value: 7e−05
Number of genes in complex: 24
Top coordinated expressed tissues: beta, islet, Testis

Node color based on: Islet biology gene sets

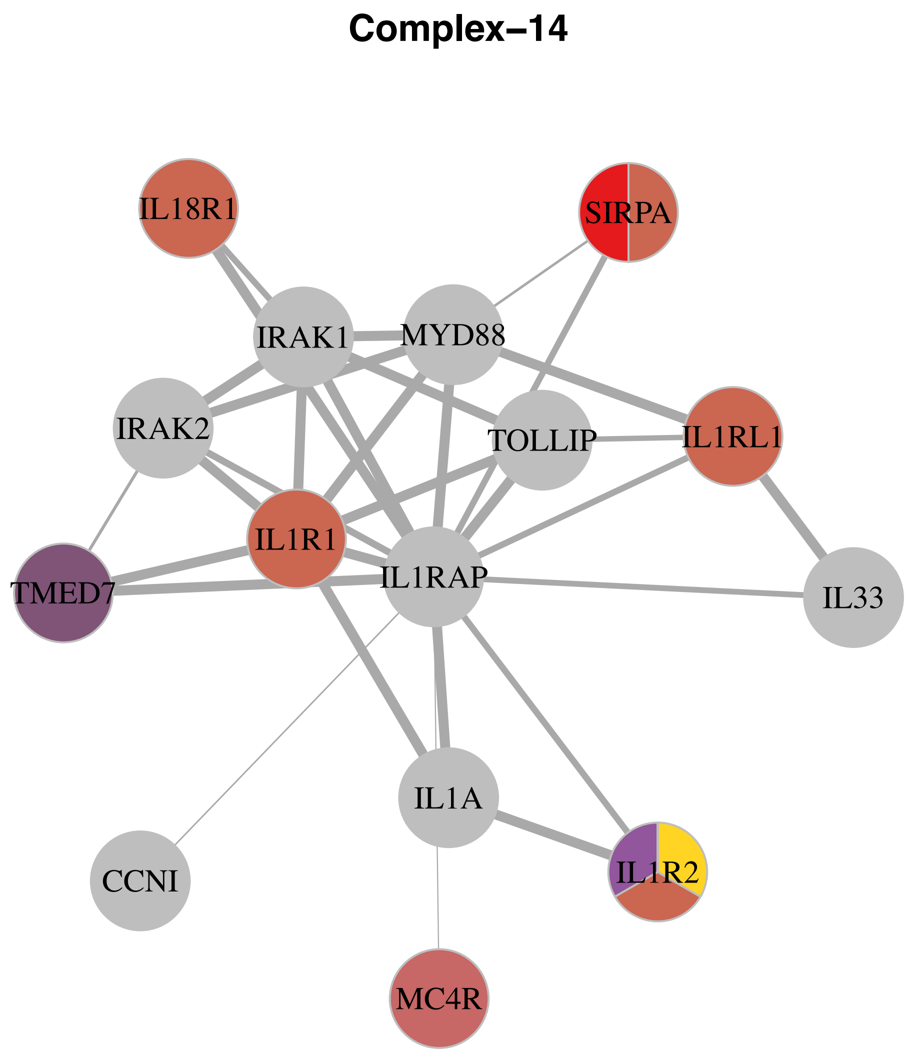

Emperical P−value: 9e−05
Number of genes in complex: 15
Top coordinated expressed tissues: Fallopian Tube, Nerve, Blood Vessel

Node color based on: Islet diabetic phenotype gene sets

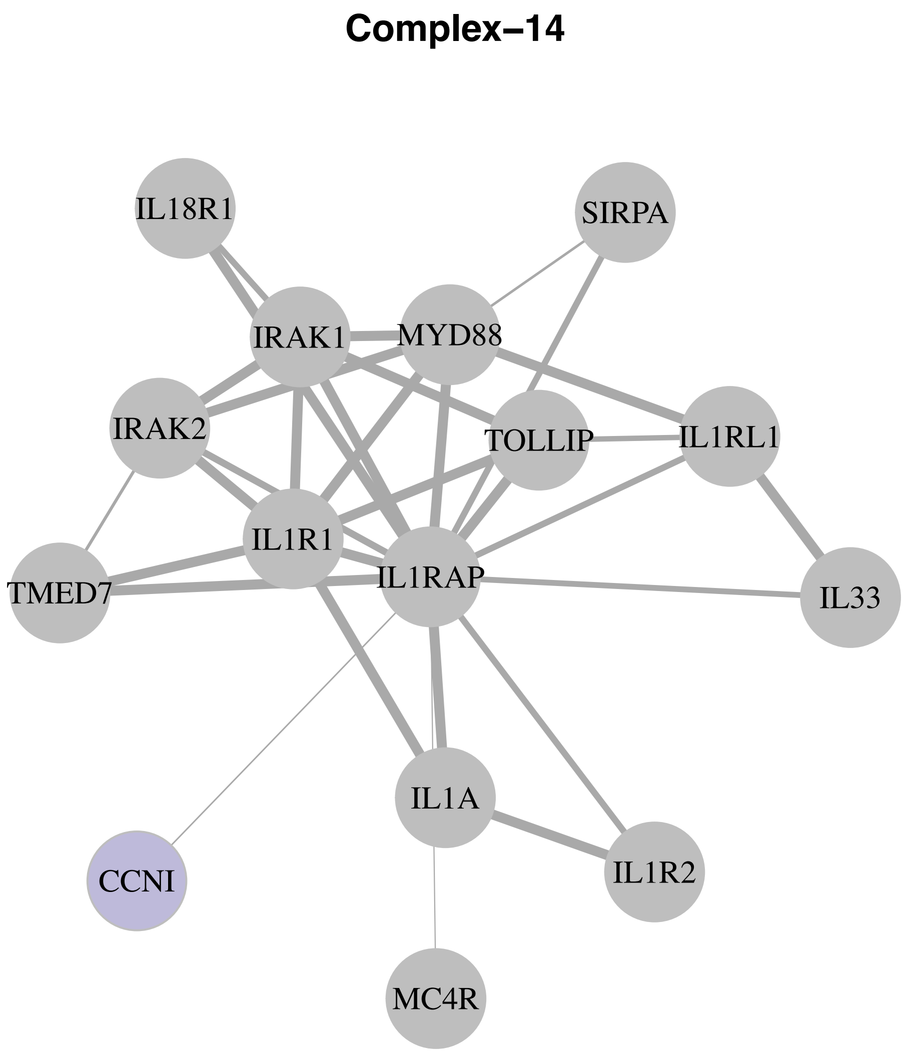

Emperical P−value: 9e−05
Number of genes in complex: 15
Top coordinated expressed tissues: Fallopian Tube, Nerve, Blood Vessel

Node color based on: Islet biology gene sets

Complex−15

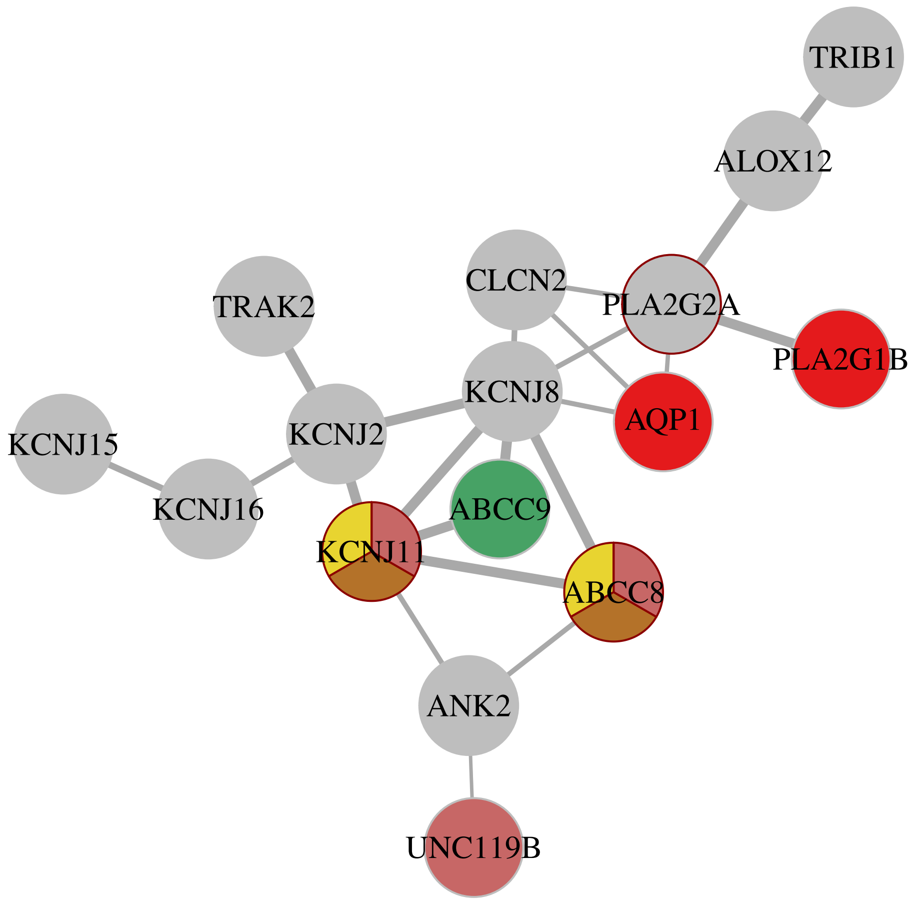

Emperical P−value: 9e−05
Number of genes in complex: 16
Top coordinated expressed tissues: Salivary Gland, islet, Bone Marrow

Node color based on: Islet diabetic phenotype gene sets

Complex−15

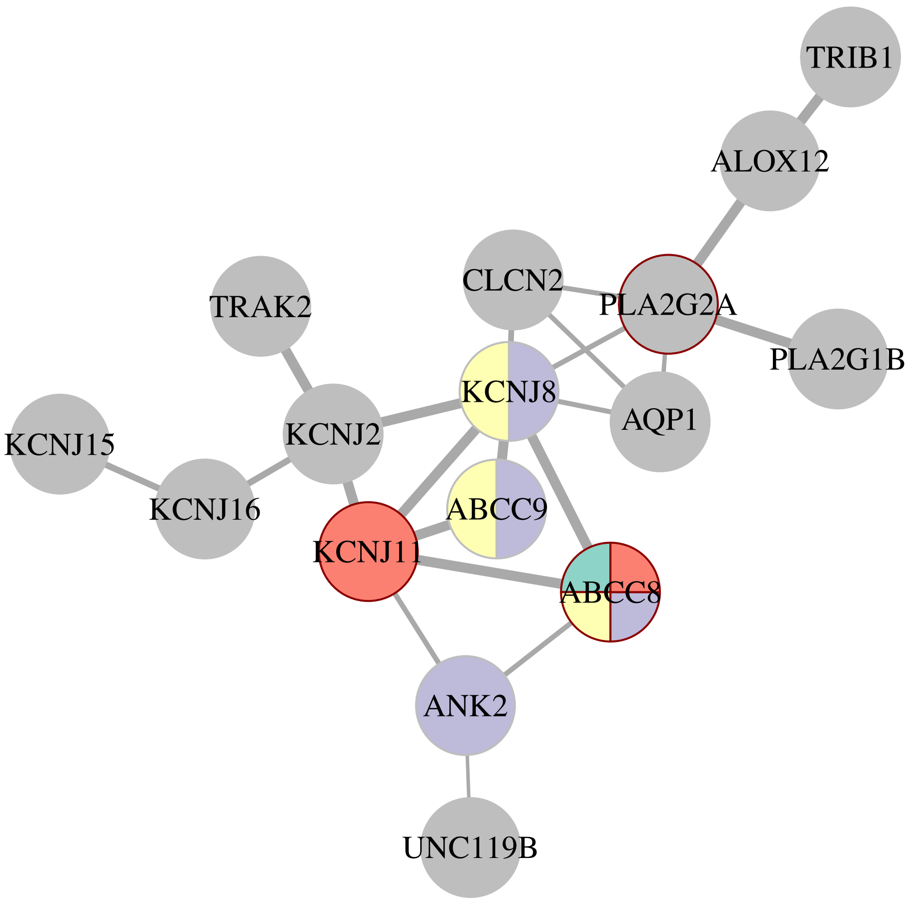

Emperical P−value: 9e−05
Number of genes in complex: 16
Top coordinated expressed tissues: Salivary Gland, islet, Bone Marrow

Node color based on: Islet biology gene sets

Complex−16

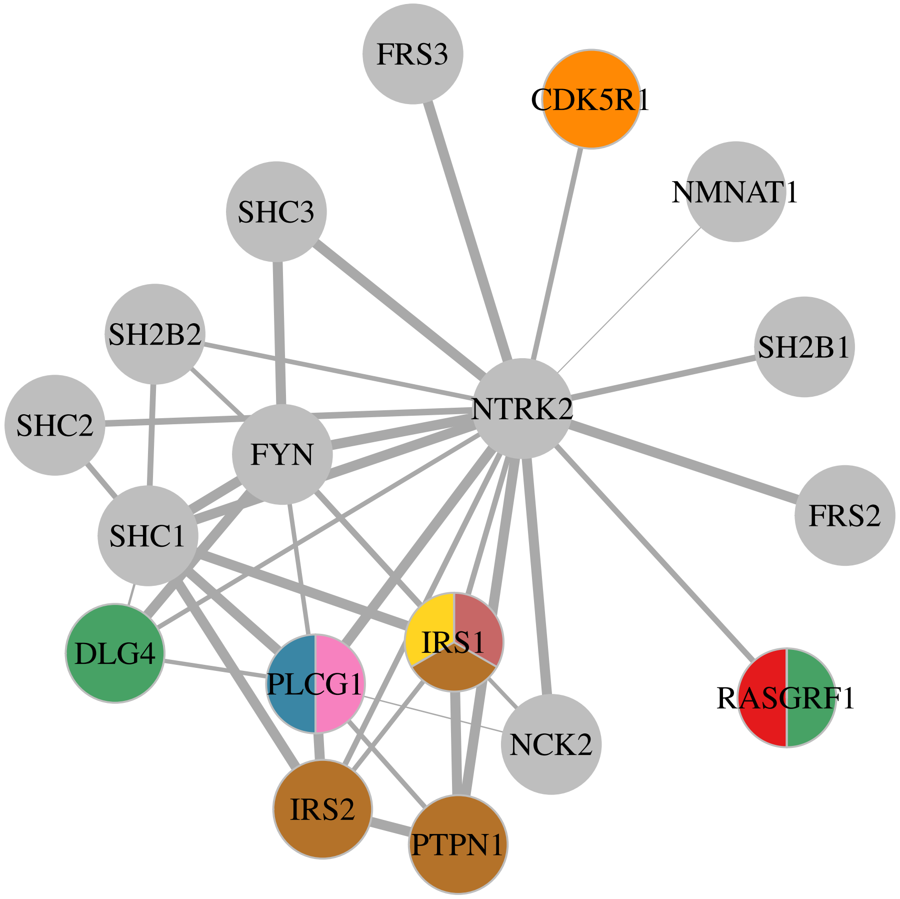

Emperical P−value: 1e−04
Number of genes in complex: 18
Top coordinated expressed tissues: Stomach, Vagina, Colon

Node color based on: Islet diabetic phenotype gene sets

Complex−16

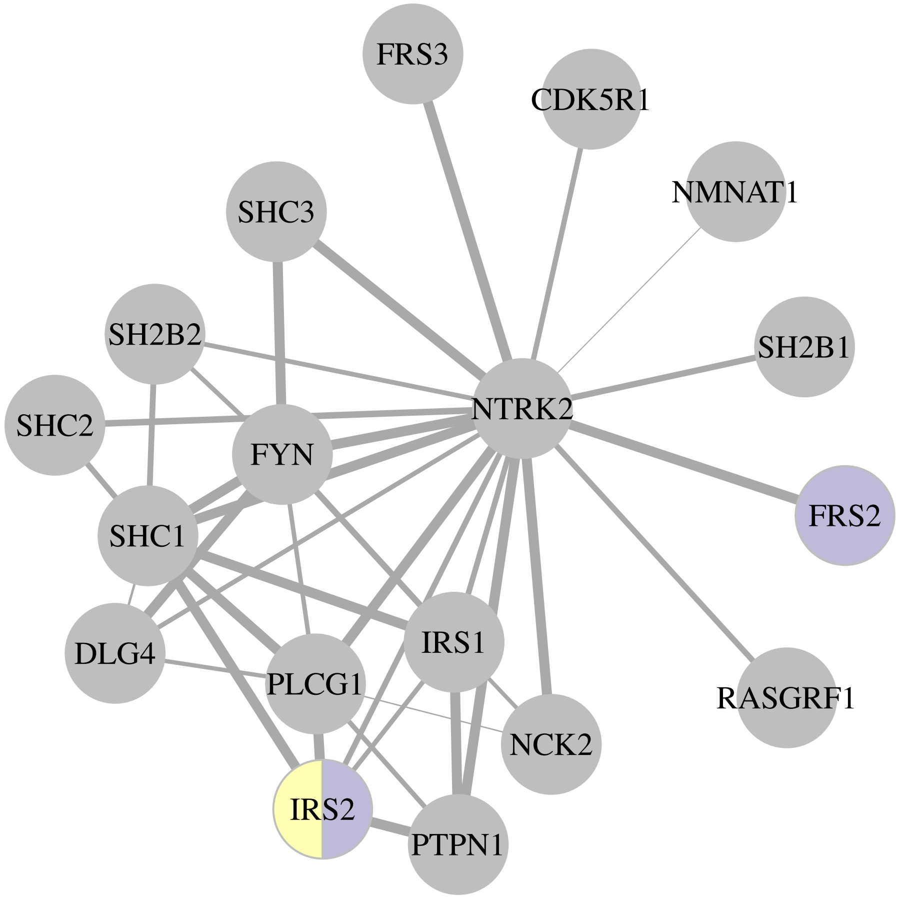

Emperical P−value: 1e−04
Number of genes in complex: 18
Top coordinated expressed tissues: Stomach, Vagina, Colon

Node color based on: Islet biology gene sets

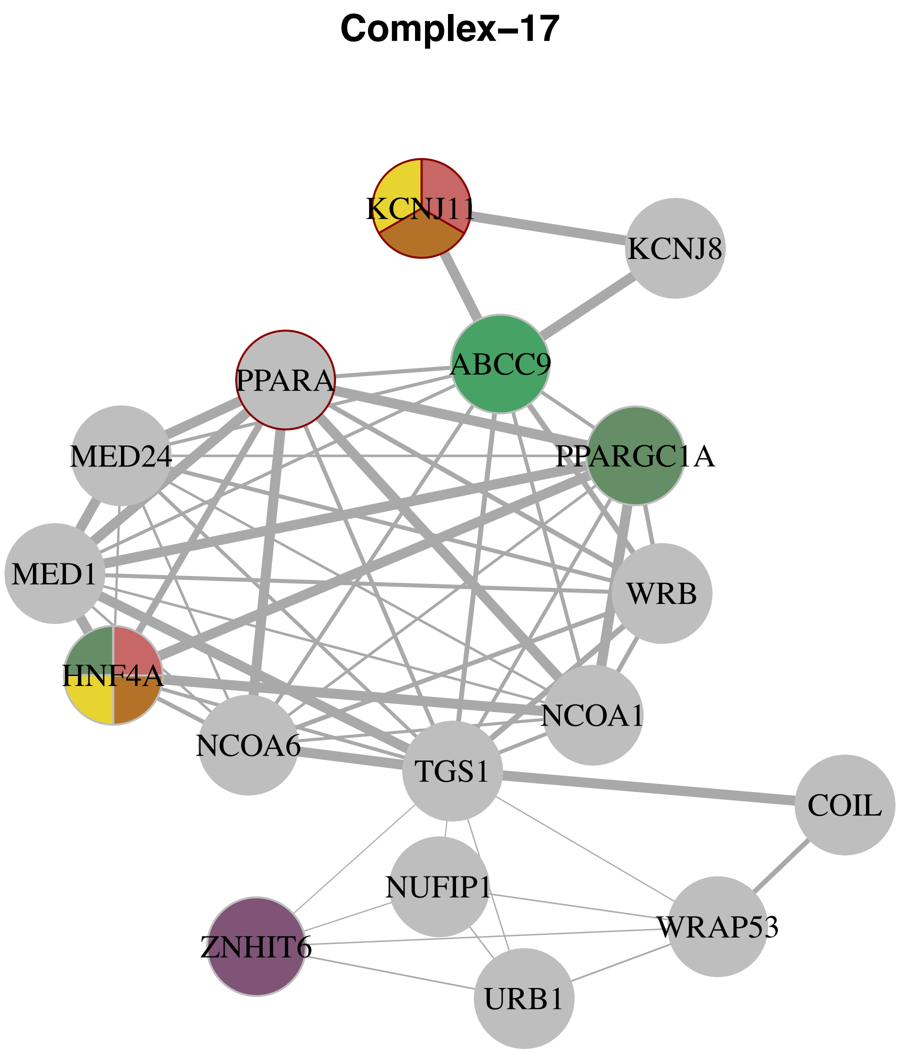

Emperical P−value: 1e−04
Number of genes in complex: 17
Top coordinated expressed tissues: islet, Vagina, Cervix Uteri

Node color based on: Islet diabetic phenotype gene sets

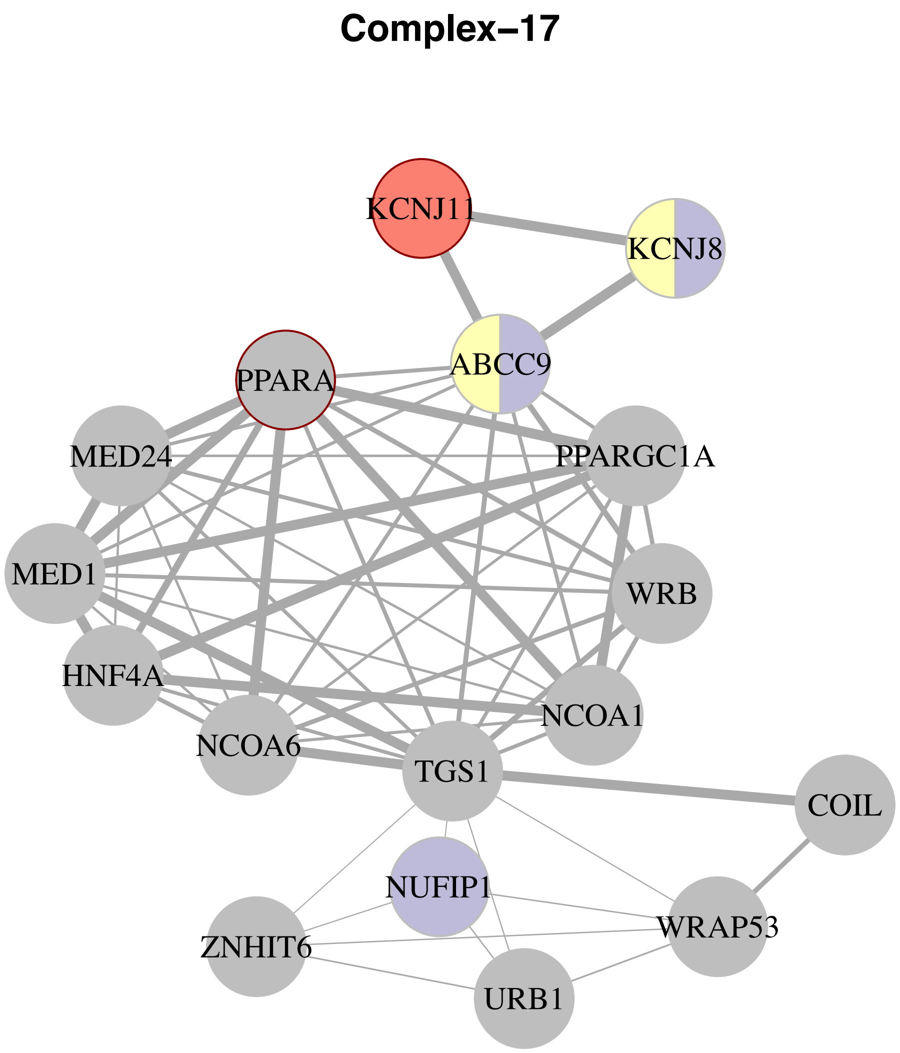

Emperical P−value: 1e−04
Number of genes in complex: 17
Top coordinated expressed tissues: islet, Vagina, Cervix Uteri

Node color based on: Islet biology gene sets

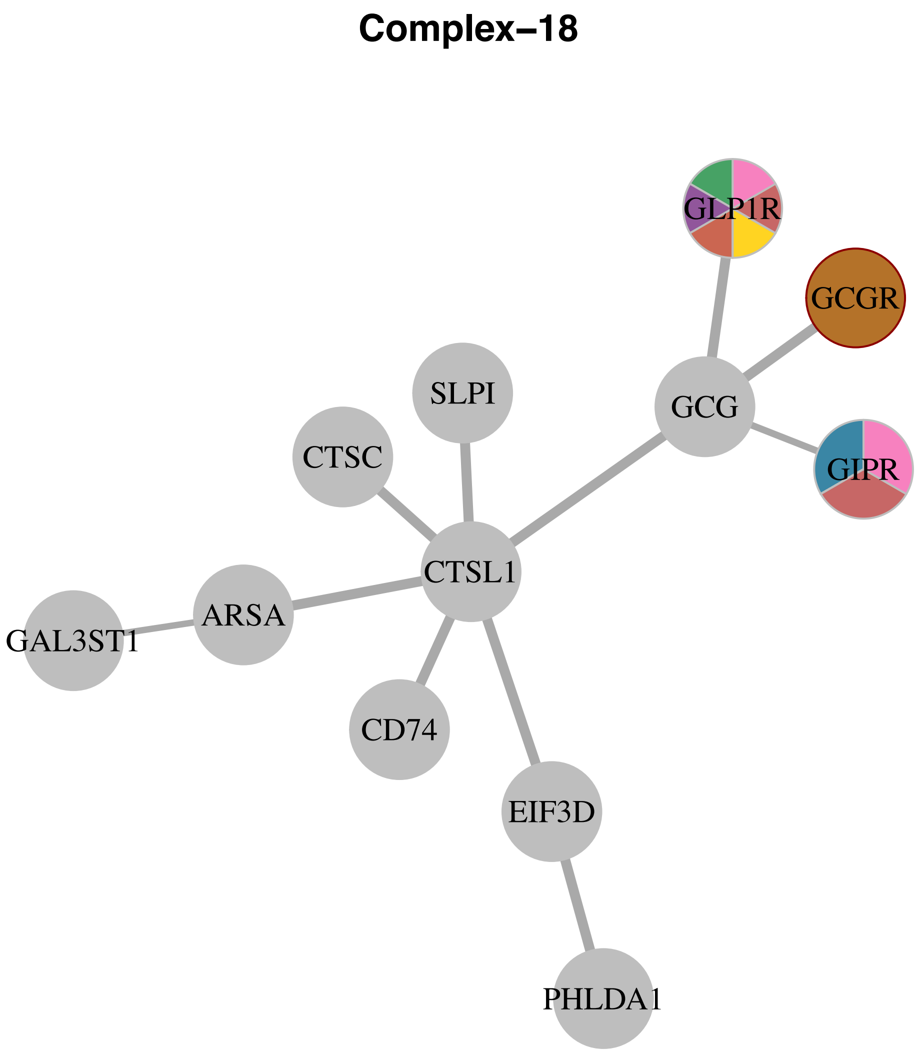

Emperical P−value: 0.00011
Number of genes in complex: 12
Top coordinated expressed tissues: Blood Vessel, Nerve, Thyroid

Node color based on: Islet diabetic phenotype gene sets

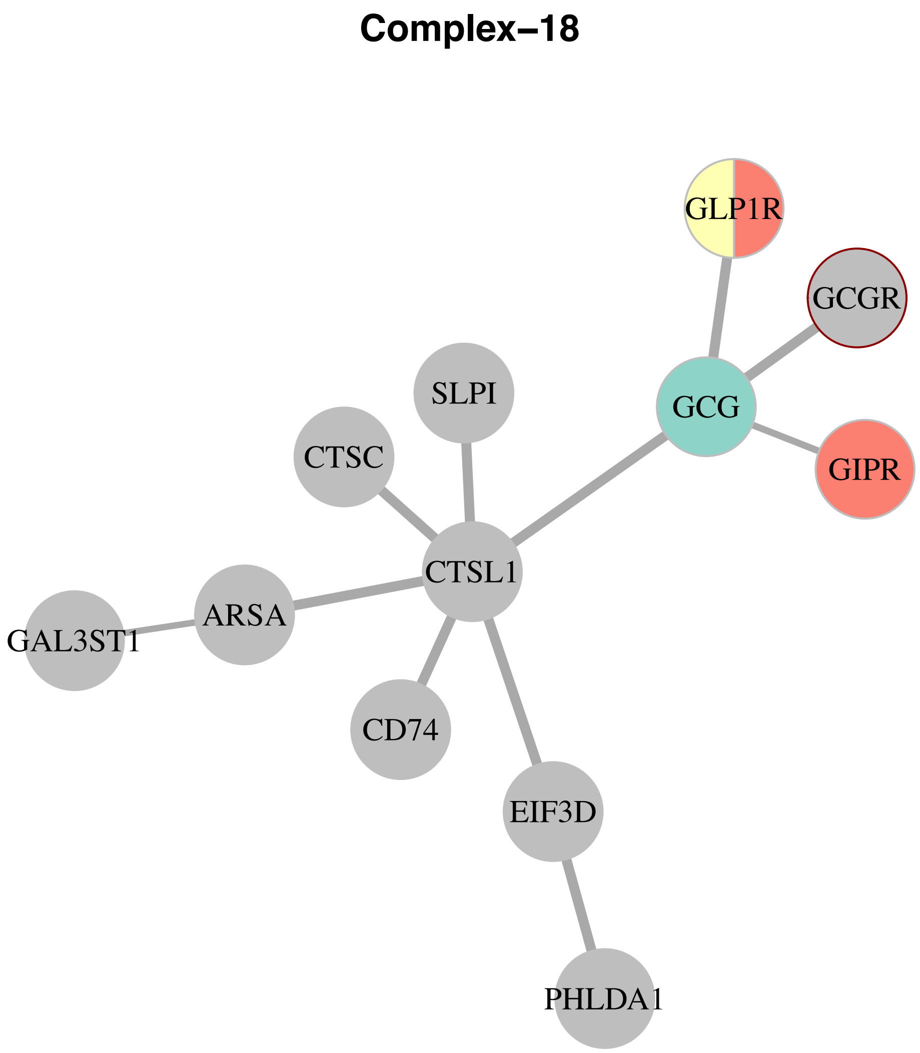

Emperical P−value: 0.00011
Number of genes in complex: 12
Top coordinated expressed tissues: Blood Vessel, Nerve, Thyroid

Node color based on: Islet biology gene sets

Complex−19

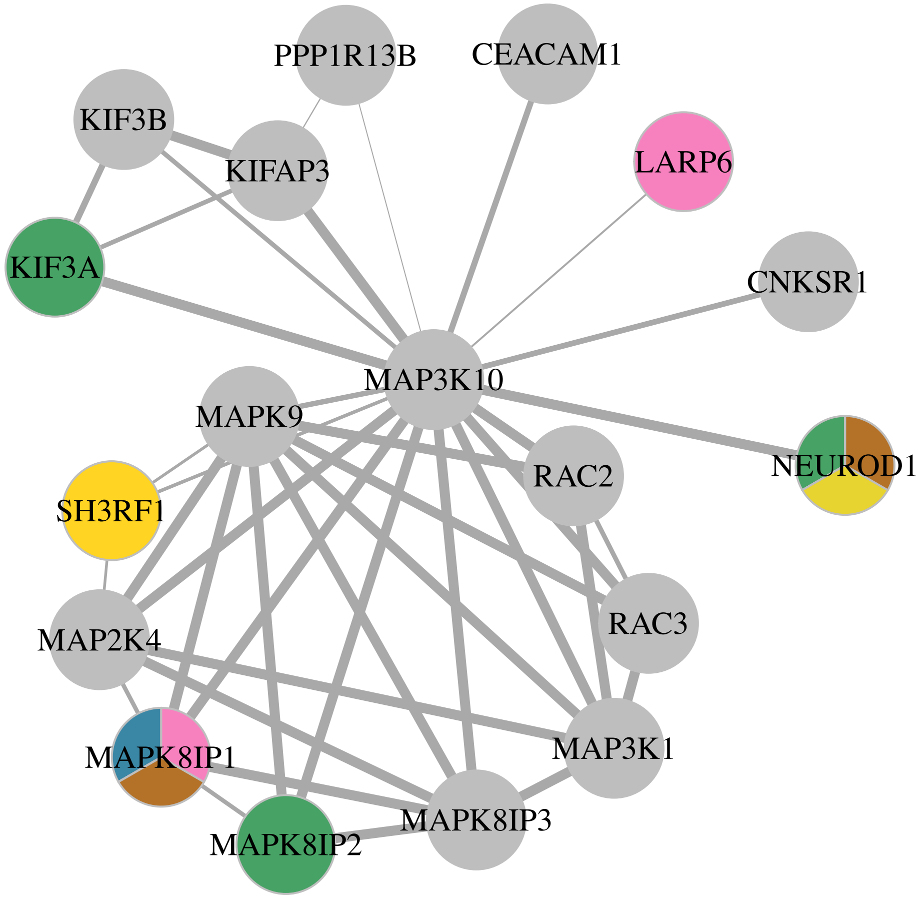

Emperical P−value: 0.00011
Number of genes in complex: 18
Top coordinated expressed tissues: Adrenal Gland, Prostate, Liver

Node color based on: Islet diabetic phenotype gene sets

Complex−19

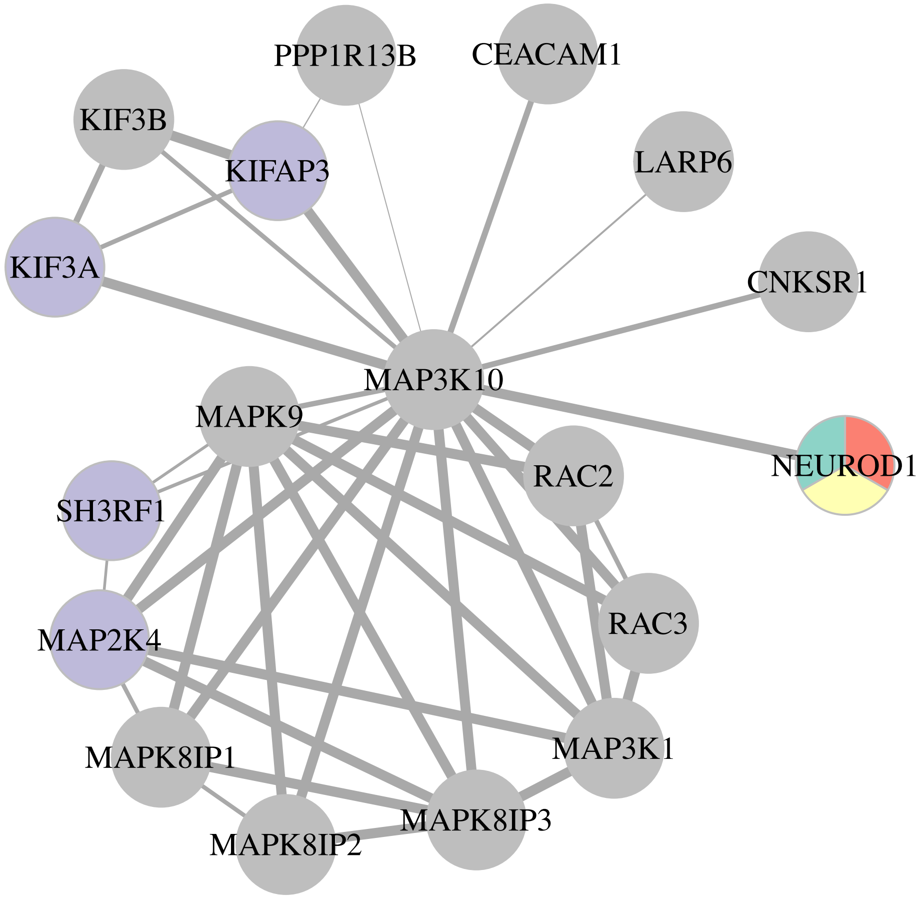

Emperical P−value: 0.00011
Number of genes in complex: 18
Top coordinated expressed tissues: Adrenal Gland, Prostate, Liver

Node color based on: Islet biology gene sets

Complex–20

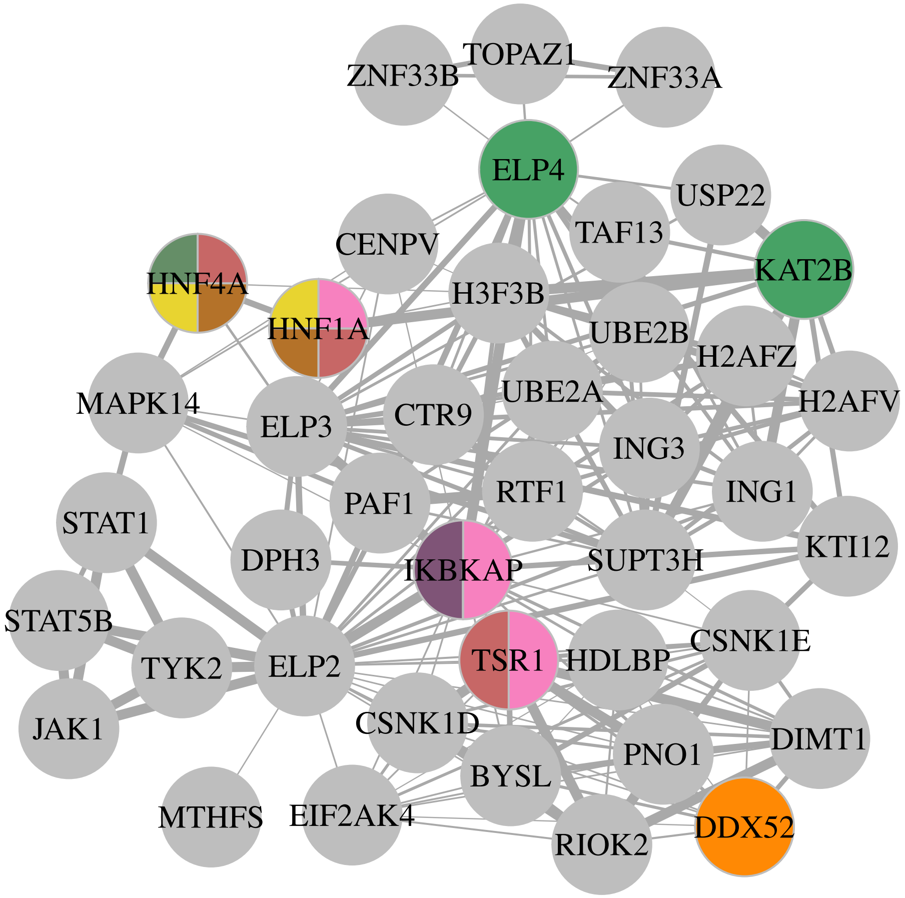

Emperical P–value: 0.00012
Number of genes in complex: 42
Top coordinated expressed tissues: Brain, Heart, Cervix Uteri

Node color based on: Islet diabetic phenotype gene sets

Complex–20

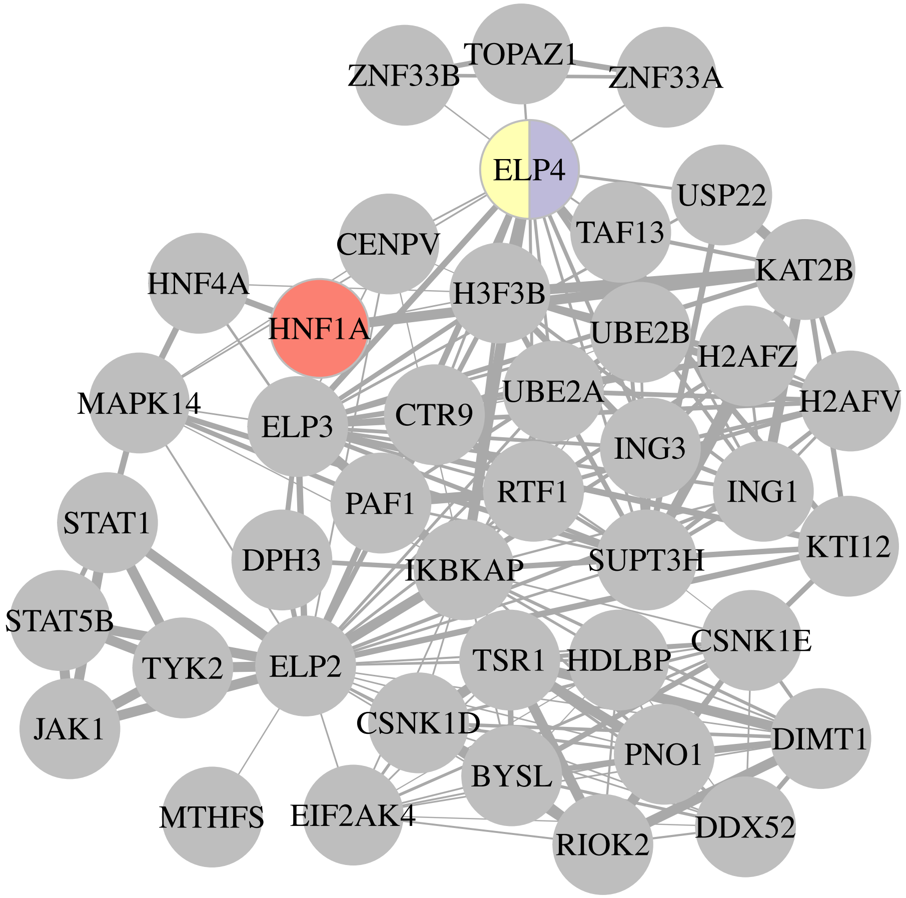

Emperical P–value: 0.00012
Number of genes in complex: 42
Top coordinated expressed tissues: Brain, Heart, Cervix Uteri

Node color based on: Islet biology gene sets

Complex−21

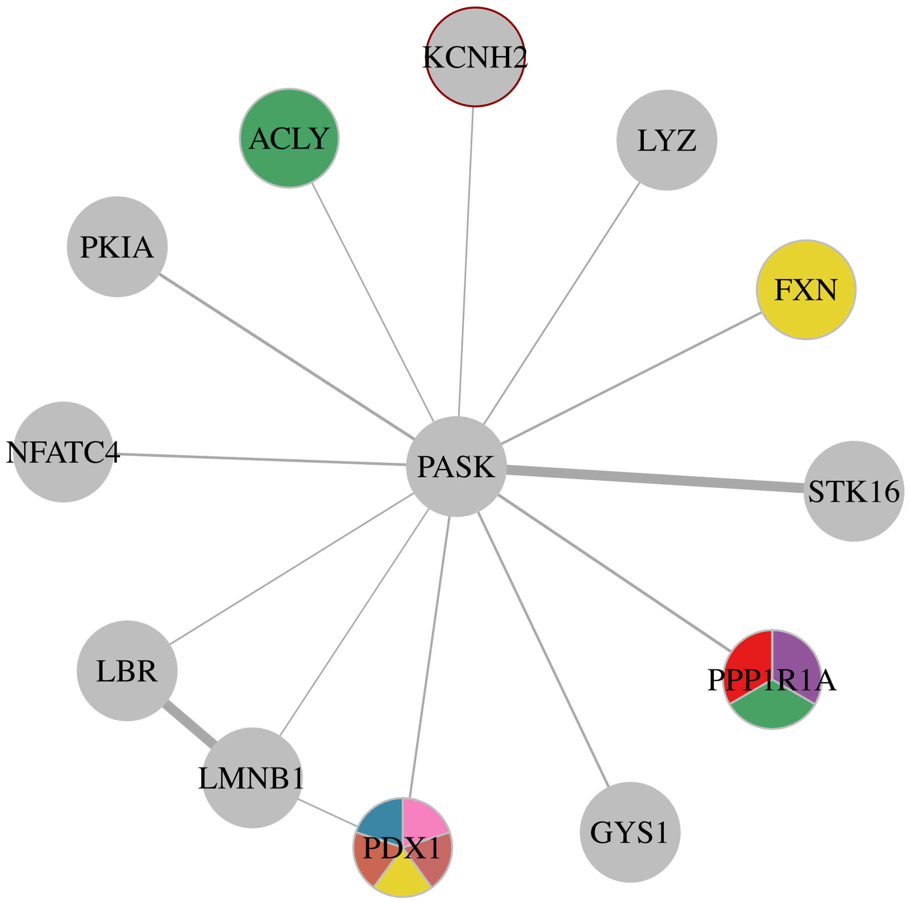

Emperical P−value: 0.00018
Number of genes in complex: 13
Top coordinated expressed tissues: Pituitary, Spleen, Liver

Node color based on: Islet diabetic phenotype gene sets

Complex−21

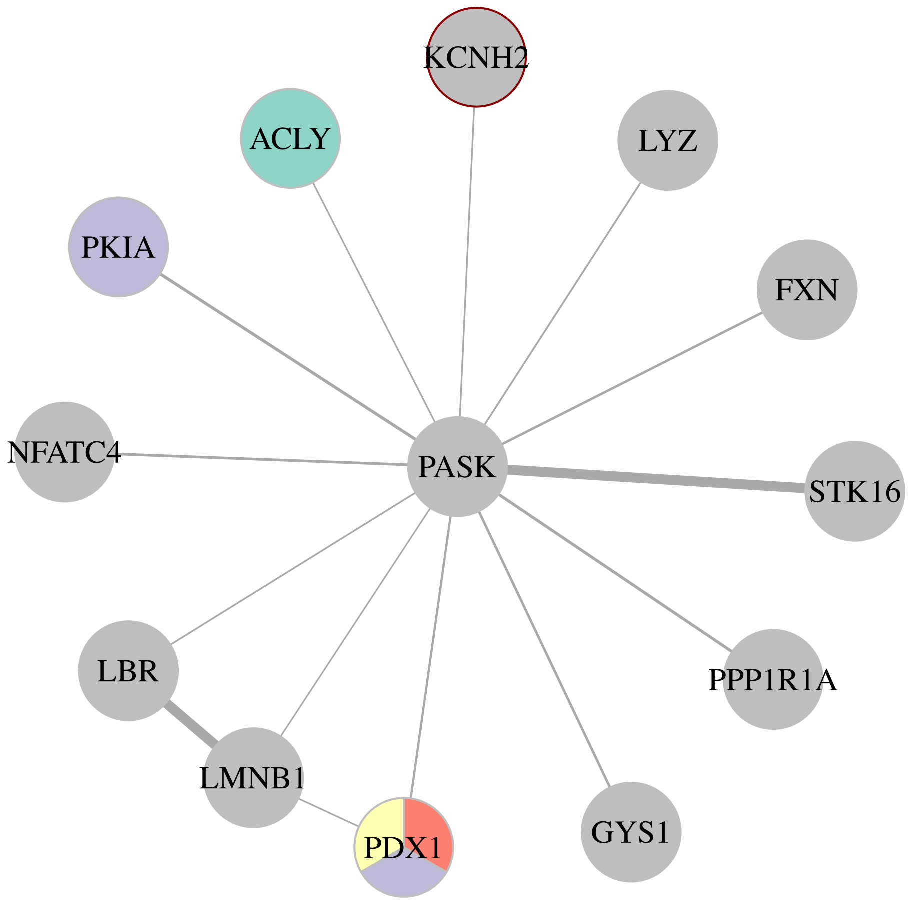

Emperical P−value: 0.00018
Number of genes in complex: 13
Top coordinated expressed tissues: Pituitary, Spleen, Liver

Node color based on: Islet biology gene sets

Complex−22

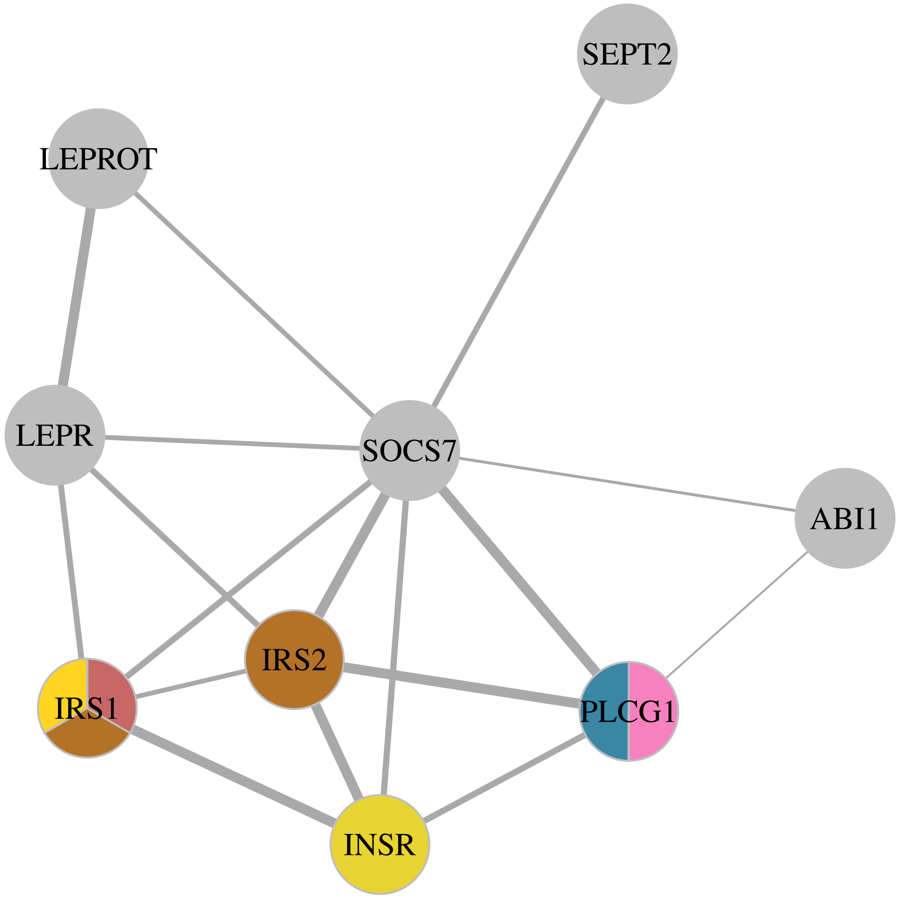

Emperical P−value: 2e−04
Number of genes in complex: 9
Top coordinated expressed tissues: Ovary, nonbeta, Colon

Node color based on: Islet diabetic phenotype gene sets

Complex−22

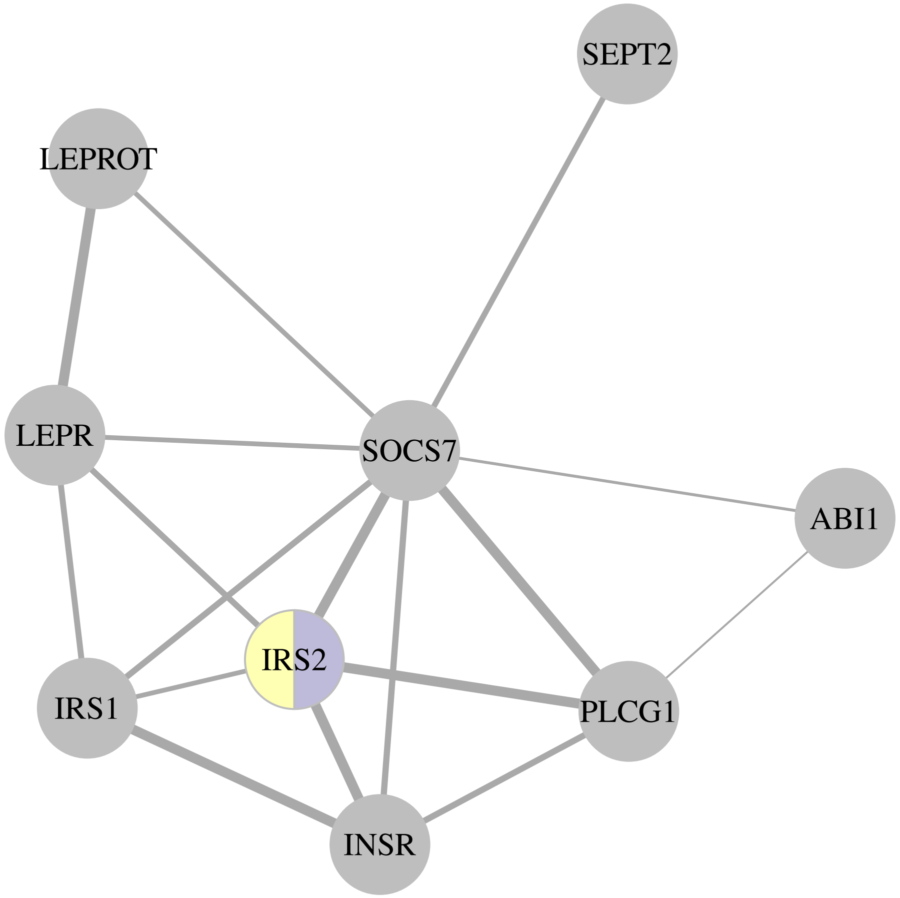

Emperical P−value: 2e−04
Number of genes in complex: 9
Top coordinated expressed tissues: Ovary, nonbeta, Colon

Node color based on: Islet biology gene sets

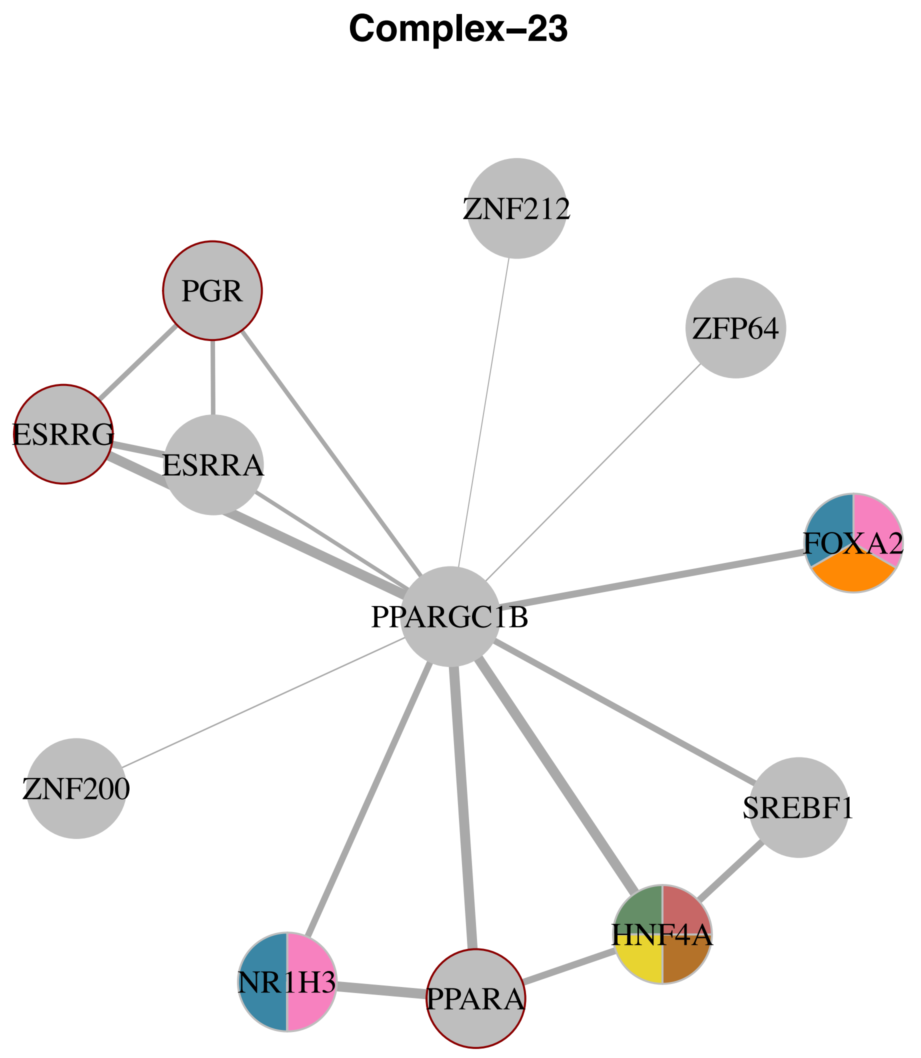

Emperical P−value: 0.00024
Number of genes in complex: 12
Top coordinated expressed tissues: Skin, beta, Colon

Node color based on: Islet diabetic phenotype gene sets

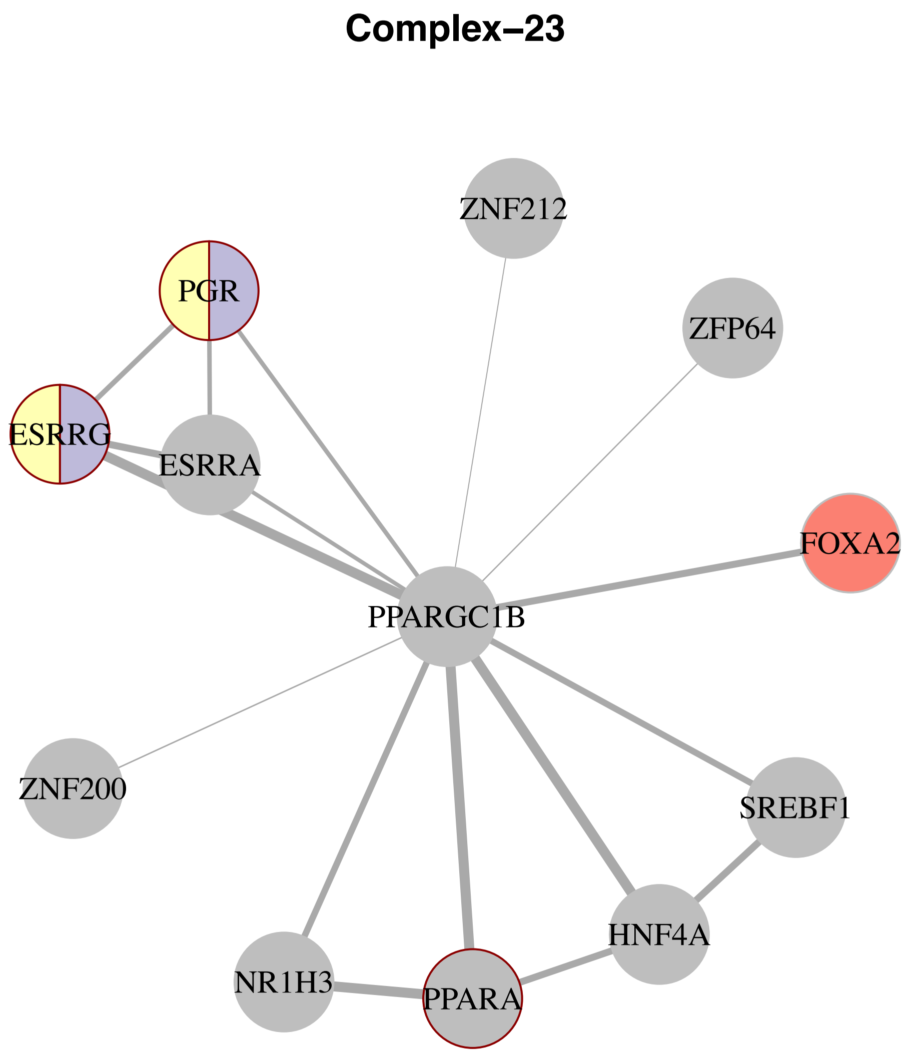

Emperical P−value: 0.00024
Number of genes in complex: 12
Top coordinated expressed tissues: Skin, beta, Colon

Node color based on: Islet biology gene sets

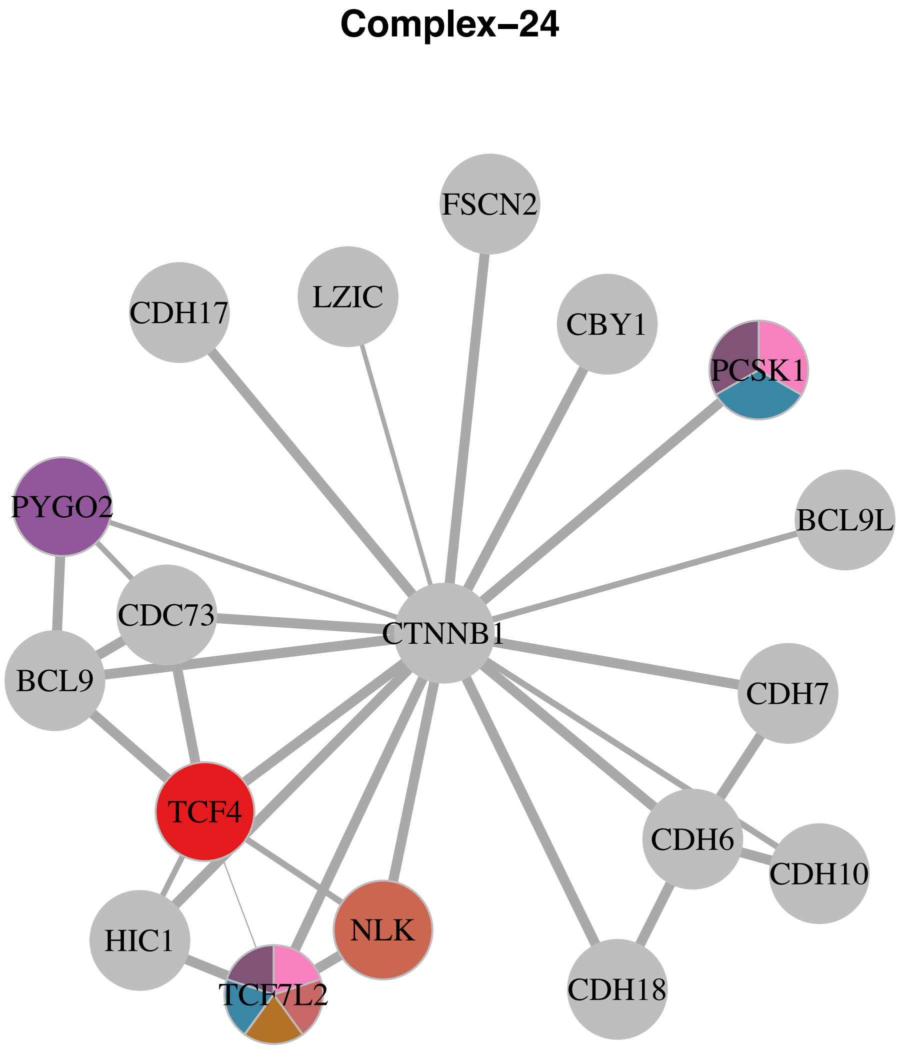

Emperical P−value: 0.00028
Number of genes in complex: 18
Top coordinated expressed tissues: Cervix Uteri, Vagina, Stomach

Node color based on: Islet diabetic phenotype gene sets

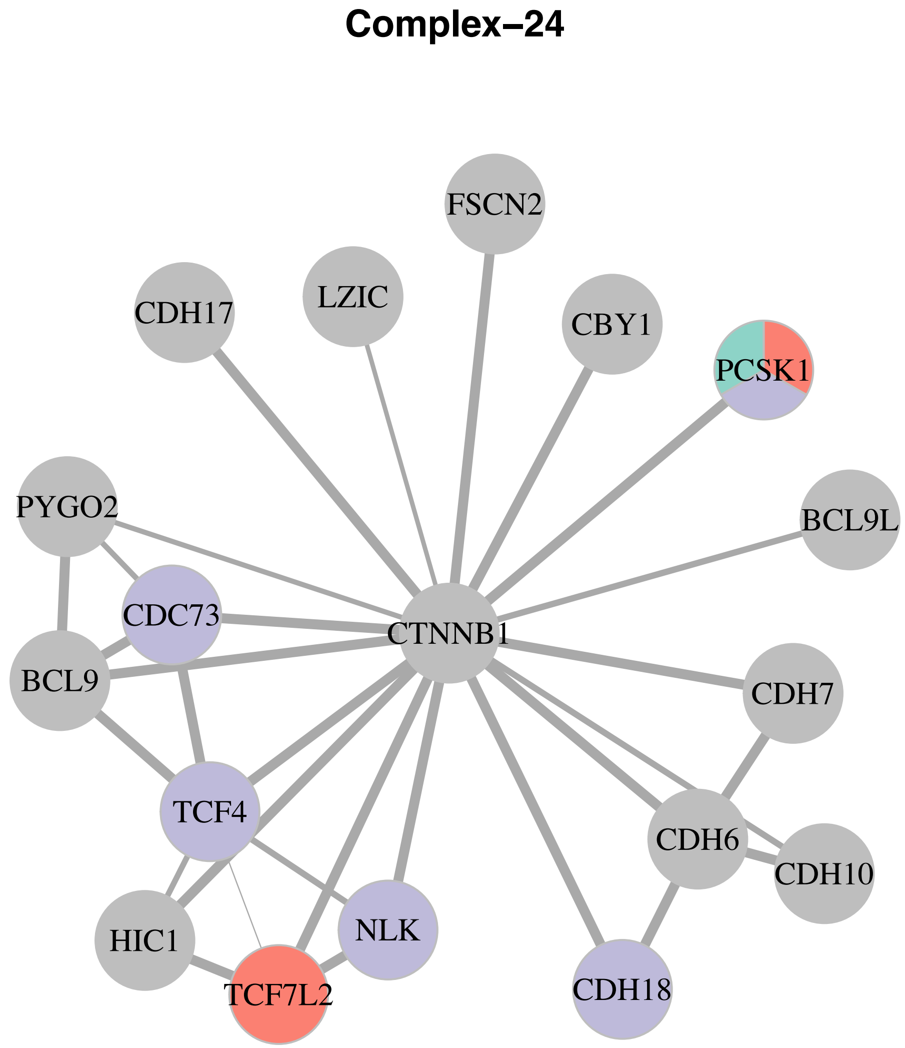

Emperical P−value: 0.00028
Number of genes in complex: 18
Top coordinated expressed tissues: Cervix Uteri, Vagina, Stomach

Node color based on: Islet biology gene sets
