# Supplementary figures and images for: Pancreatic Islet Protein Complexes and Their Dysregulation in Type 2 Diabetes
Source: Front Genet. 2017 Apr 20;8:43. doi: 10.3389/fgene.2017.00043 (PMC5397424; doi:10.3389/fgene.2017.00043)

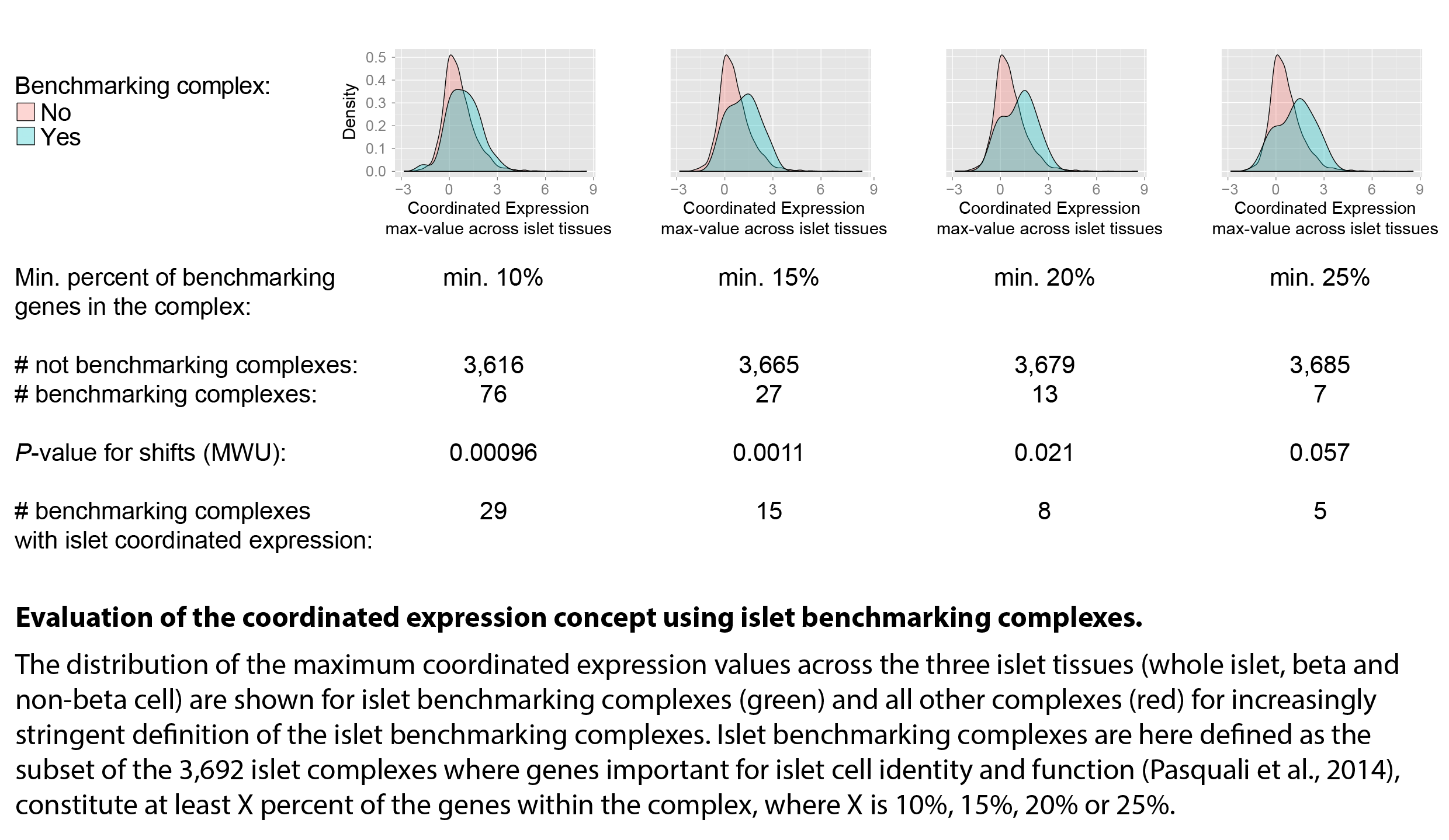

Supplement: Supplementary file 4 [file Image1.PNG]

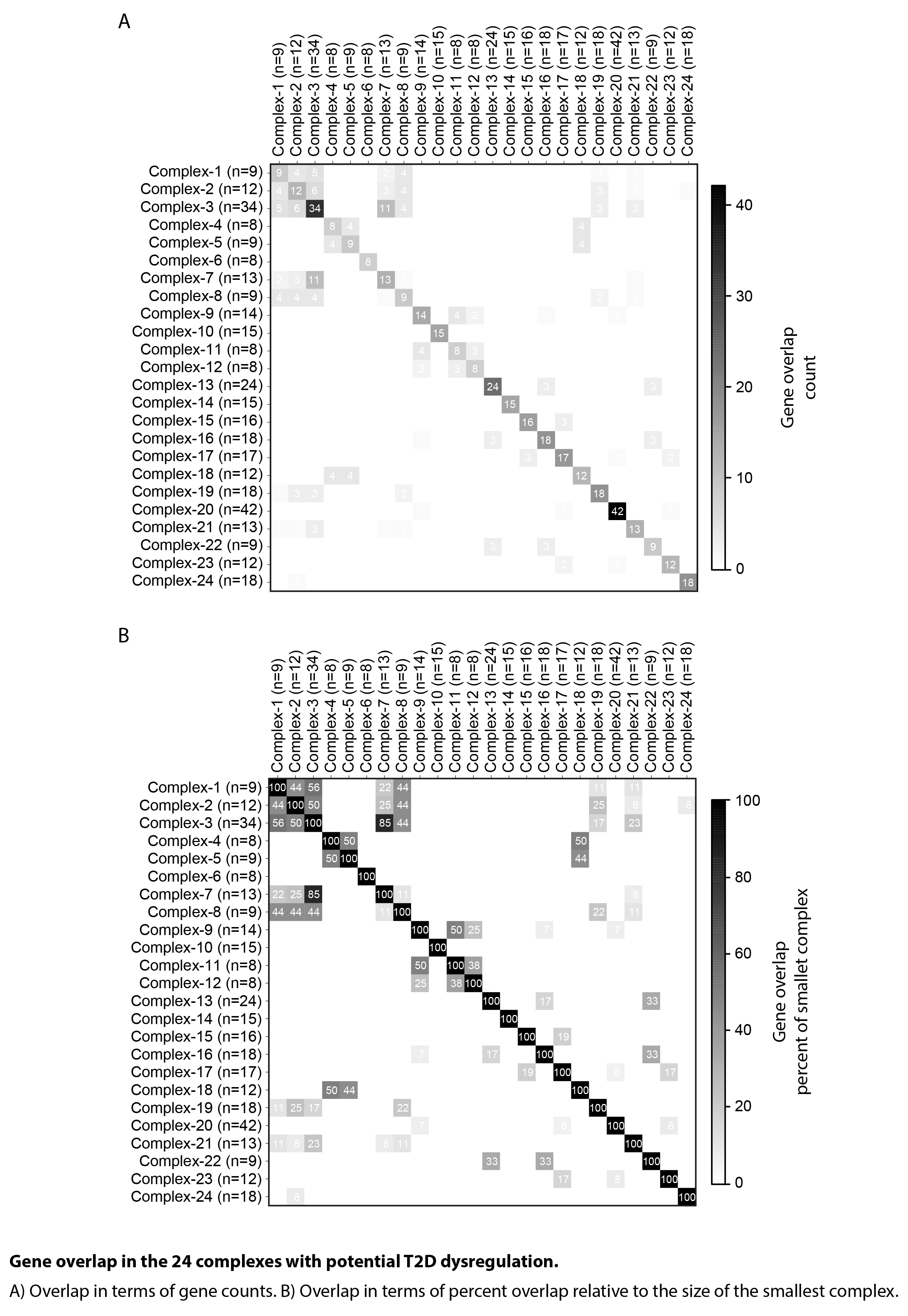

Supplement: Supplementary file 5 [file Image2.png]

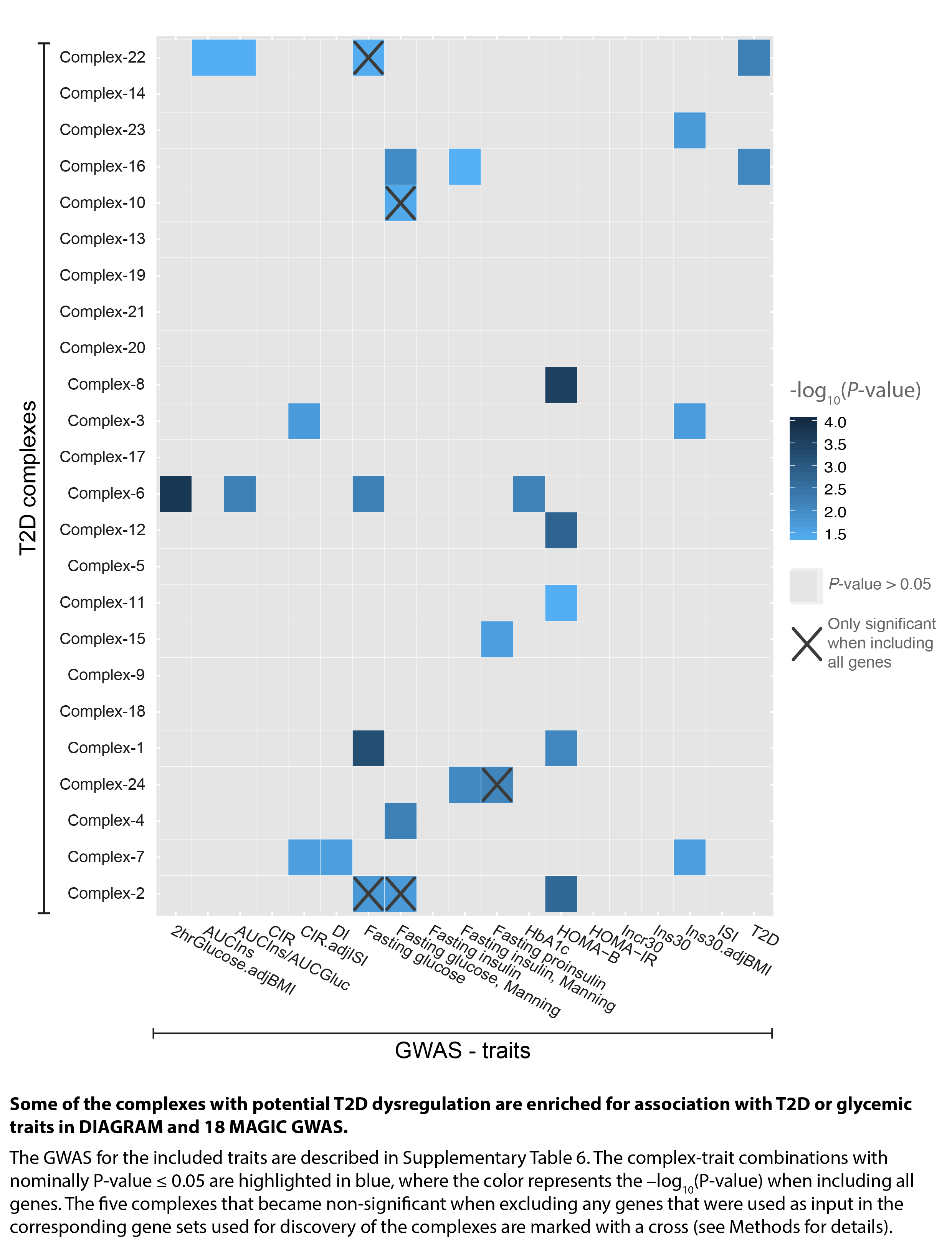

Supplement: Supplementary file 6 [file Image3.png]
